# Both activated and less‐activated regions identified by functional MRI reconfigure to support task executions

**DOI:** 10.1002/brb3.893

**Published:** 2017-12-20

**Authors:** Nianming Zuo, Zhengyi Yang, Yong Liu, Jin Li, Tianzi Jiang

**Affiliations:** ^1^ Brainnetome Center Institute of Automation Chinese Academy of Sciences Beijing China; ^2^ National Laboratory of Pattern Recognition Institute of Automation Chinese Academy of Sciences Beijing China; ^3^ CAS Center for Excellence in Brain Science and Intelligence Technology Institute of Automation Chinese Academy of Sciences Beijing China; ^4^ Key Laboratory for NeuroInformation of the Ministry of Education School of Life Science and Technology University of Electronic Science and Technology of China Chengdu China; ^5^ The Queensland Brain Institute University of Queensland Brisbane QLD Australia; ^6^ University of Chinese Academy of Sciences Beijing China

**Keywords:** activation, brain network, functional connectivity, functional magnetic resonance imaging, network reconfiguration

## Abstract

**Introduction:**

Functional magnetic resonance imaging (fMRI) has become very important for noninvasively characterizing BOLD signal fluctuations, which reflect the changes in neuronal firings in the brain. Unlike the activation detection strategy utilized with fMRI, which only emphasizes the synchronicity between the functional nodes (activated regions) and the task design, brain connectivity and network theory are able to decipher the interactive structure across the entire brain. However, little is known about whether and how the activated/less‐activated interactions are associated with the functional changes that occur when the brain changes from the resting state to a task state. What are the key networks that play important roles in the brain state changes?

**Methods:**

We used the fMRI data from the Human Connectome Project S500 release to examine the changes of network efficiency, interaction strength, and fractional modularity contributions of both the local and global networks, when the subjects change from the resting state to seven different task states.

**Results:**

We found that, from the resting state to each of seven task states, both the activated and less‐activated regions had significantly changed to be in line with, and comparably contributed to, a global network reconfiguration. We also found that three networks, the default mode network, frontoparietal network, and salience network, dominated the flexible reconfiguration of the brain.

**Conclusions:**

This study shows quantitatively that contributions from both activated and less‐activated regions enable the global functional network to respond when the brain switches from the resting state to a task state and suggests the necessity of considering large‐scale networks (rather than only activated regions) when investigating brain functions in imaging cognitive neuroscience.

## 1 INTRODUCTION

When the brain is occupied by a cognitively demanding task, such as responding to auditory, visual, or other stimuli, the neuronal firing and signal processing of the affected functional regions are upregulated, resulting in an increased cerebral metabolic rate, as indicated by increased oxygen uptake. Two physiological changes occur during this process: increased local cerebral blood flow (CBF) and changes in oxygenation, as measured by the blood oxygen level‐dependent (BOLD) contrast (Glover, 2011; Logothetis, 2008). Although both of these fluctuations can be detected by magnetic resonance imaging (MRI) techniques, the latter, the BOLD contrast, is more widely used to characterize activation maps of the brain because of its comparably short acquisition time and high sensitivity (Glover, 2011). Therefore, the BOLD contrast is used in conventional functional MRI (fMRI) to characterize brain activity. To define an activation map based on fMRI, two mental conditions usually are compared statistically: One is the experimental condition (the task), which causes specific functional regions to be activated, and the other is the baseline state, which is used to exclude the regions identified as not specific to the task (Stark & Squire, 2001; Voyvodic, Petrella, & Friedman, 2009).

In the literature, the activation region identified as described above was usually chosen as the region of interest (ROI), which was regarded as the main actor when executing a specific task. Subsequently, a functional analysis was conducted. A large number of findings based on this procedure have been reported in recent decades (Chu et al., 2015; Poldrack, 2007). However, the fact that these studies showed both areas that were activated and those that were not does not mean that the so‐called nonactivated regions were “inactive” or had nothing to do with the tasks. In this traditional type of study, the “less‐activated” regions (L‐Act), as suggested by Yamashita and colleagues (Yamashita, Kawato, & Imamizu, 2015), were just considered to be the complementary set to the activated regions (Act), but their synchronicity with the task stimulus was considered to be statistically insignificant. In fact, studies have revealed that the brain consumes a small additional portion of energy in an attention‐demanding task state beyond what is used in the resting state (Raichle, 2009, 2010). Although the change evoked by the task stimulus is subtle (Cole, Bassett, Power, Braver, & Petersen, 2014), the reconfiguration of the network architecture from resting state to task state has received increasing attention. For example, consistent findings have indicated that the network efficiency increases and modularity decreases when the brain changes from the resting state to a cognitively demanding task (Hearne, Cocchi, Zalesky, & Mattingley, 2017; Wen et al., 2015). Additionally, these changes can predict the task complexity (Wen et al., 2015), individual intelligence and cognitive capability (Schultz & Cole, 2016), and even the aging process (Gallen, Turner, Adnan, & D'Esposito, 2016). Therefore, it is reasonable to question the following: (1) Whether and how are the activated/less‐activated interactions associated with the functional changes that occur when the brain changes from the resting state to a task state? (2) To what degree do the less‐activated regions contribute to network changes in global reconfiguration and is this comparable to the contribution of the activated regions? (3) What are the key networks that play important roles in brain state changes?

Unlike the activation detection strategy, which only emphasizes the synchronicity between the functional nodes and the task design, as described above, functional connectivity and brain network theory are able to decipher the interactive structure across the entire brain (Bullmore & Sporns, 2012; Sporns, 2011). Therefore, to try to answer the above questions, we investigated fMRI data from a dataset released by the Human Connectome Project (HCP) (Van Essen et al., 2013) that included both resting state and task data in seven tasks (See Table 1 for a list.) from a functional network point of view. First, we used three network metrics, that is, interaction strength, modularity, and the efficiency index, to measure network reconfigurations within and between the Act, L‐Act, and global networks. Then, we proposed “fractional modularity” as a measure of the modular contribution to the global network for a given region and applied it to both the Act and L‐Act regions to compare their contributions to the changes in global network modularity. Finally, we examined which functional subnetworks reconfigure the most intensively when the brain changes from the resting state to the task state. All the source code in this study (in MATLAB) and the activation maps are available in https://github.com/nmzuo/Act-L-Act-network.

**Table 1:** The number of scanning frames and the run duration for each task fMRI data collection in HCP data (adapted from http://protocols.humanconnectome.org/HCP/3T/imaging-protocols.html)

Task	Runs	Frames per run	Run duration (min:s)
Working memory	2	405	5:01
Gambling	2	253	3:12
Motor	2	284	3:34
Language	2	316	3:57
Social cognition	2	274	3:27
Relational processing	2	232	2:56
Emotion processing	2	176	2:16

## 2 MATERIALS AND METHODS

### 2.1 Data acquisitions and preprocessing

The HCP data S500 release was used in this study. There were 512 subjects’ records in the data we obtained from the HCP. In the current work, only the fMRI data were utilized. Some data were excluded using the following criteria: (1) the data reported on the Known‐Issues page of the HCP website, htttps://wiki.humanconnectome.org/display/PublicData/HCP+Data+Release+Updates%3A+Known+Issues+and+Planned+fixes; (2) data that did not have complete time points as indicated by the correct number of frames for each mental state (see Table 1); (3) data that did not have a full EV record to accompany the fMRI data; and (4) data acquired without left–right (LR) or right–left (RL) phase encoding. In the end, 453 subjects (age 29.1 ± 3.5 years, 188 male) were used in the subsequent analyses. The task datasets contained seven conventional tasks, including working memory (WM), gambling, motor, language, social cognition, relational processing, and emotion processing, each of which had been designed to activate specific functional regions (Barch et al., 2013). To ensure a large population for this study, subjects who had biological relationships with others in the HCP data release were retained in our dataset (Cole, Bassett et al., 2014; Smith et al., 2015).

To account for the influence of the phase encoding direction during MRI scanning, both left–right (LR) and right–left (RL) phase encodings were adopted in two separate scan sessions in the HCP. In this study, we repeated the analysis on three different dataset combinations, that is, the LR dataset only, the RL dataset only, and the averaged LR/RL dataset. Considering the large number of results and figures, only the results from the averaged dataset are presented in the manuscript and the results from the other two datasets are included in the Supporting information.

The HCP dataset was partially used in our previous work (Zuo, Song, Fan, Eickhoff, & Jiang, 2016), and the descriptions of the imaging protocols and preprocessing steps used by the HCP consortium are described in Section S1. Here, we will only repeat the main descriptions and preprocessing steps. The dataset was collected on a 3T MRI Skyra scanner (Siemens, Germany) using a standard 32 channel head coil. The magnetic field produced by the coil was modeled to provide a customized distortion correction. The primary scanning parameters were as follows: repetition time (TR), 720 ms; echo time (TE), 33.1 ms; flip angle, 52°; field of view, 208 × 180 mm; slice thickness, 2.0 mm; and voxel size, 2.0‐mm isotropic cube (Van Essen et al., 2013). The HCP data were already preprocessed, well aligned, and registered to the Montreal Neurological Institute (MNI) 2‐mm standard space when we received it. The main preprocessing steps taken included (Glasser et al., 2013): (1) gradient nonlinearity distortion; (2) 6 degrees of freedom (DOF) FSL/FLIRT‐based motion correction; (3) FSL/top‐up‐based distortion correction; (4) registration to a T1 space image; and (5) FSL/FNIRT‐based registration to MNI 2‐mm space. After receiving the above preprocessed data from HCP, we further band‐pass‐filtered the data at 0.009–0.08 Hz to reduce low‐frequency drift and high‐frequency noise (Vatansever, Menon, Manktelow, Sahakian, & Stamatakis, 2015). The mean signal of the white matter and cerebrospinal fluid (CSF) and the movement parameters and its derivatives (in the Movement_parameters.txt file in HCP S500 release) were regressed out as confounding factors. Since we used a series of ROIs in this study to sample the gray matter of the entire brain (described below), a smoothing step was not applied here. In addition, global signal regression was not conducted because its use is controversial and there is no consensus about its physiological interpretation (Bassett, Yang, Wymbs, & Grafton, 2015; Mayhew et al., 2016).

### 2.2 The overall strategy of this study

**Figure 1:**
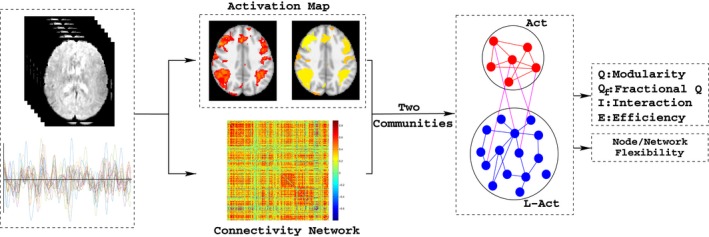
The analysis strategy in this work. First, the activated and less‐activated regions were identified based on the global fMRI time course, and the global connectivity matrices were generated. Second, the networks were partitioned by the activity and nonactivity masks. Using the above, the modularity indices for the global and for the two classes of regions were determined. Finally, the interactions between the two regions and the individual changes in these two regions were also characterized

The analysis pipeline used in this study is illustrated in Figure 1. We used four network properties to characterize the network configuration: interaction strength, modularity, efficiency, and flexibility. These properties were quantified for the resting state and all seven task states. By checking the changes in these quantities, we were able to describe the network reconfiguration when the brain changes from the resting state to a task state. Step 1: We identified the activated regions for each of the seven task states in the HCP S500 release. Hence, both the Act and L‐Act regions (the complementary set to the former) could be identified. Step 2: To examine whether the task state would change the global efficiency index in ways that contrasted to the resting state, we evaluated the network efficiency of the entire brain for both the resting and task states. Step 3: Based on the two classes of regions (Act and L‐Act) identified in Step 1, we computed the interaction strength between each pair of regions and the fractional modularity index (see below) to evaluate each regions’ contribution to the whole brain network modularity. Step 4: We computed the network efficiency separately for the two regions, using a nodal flexibility of 10 to predefine the functional subnetworks (Power et al., 2011) and, further, to examine whether certain subnetworks contributed differently to the global network reconfiguration.

### 2.3 Extracting activated and less‐activated maps

To generate the activation maps for the seven tasks, 30 unrelated subjects were chosen from the HCP S500 dataset by these criteria: (1) They did not have twins in the dataset (namely both the Twin_Stat and Zygosity labels were NotTwin). (2) They did not share a father or mother with others in the dataset. (3) They had both LR and RL data. And (4) they were aged 20–30 years. We adopted these requirements primarily to reduce the influence of bias on the entire group by removing related subjects from the subsequent analysis. To reduce the bias from the encoding direction for the activation detection, the LR/RL data of these 30 subjects were mixed to form a dataset of 60. In this study, the Act/L‐Act maps were extracted to assess their roles in each task, so the extraction strategy itself was not our primary emphasis. The sole purpose of the activation detection that we did here was to guarantee that the extracted maps based on the routine parameters are the ones that are well accepted in the literature for each task. Our goal was to dissect the functions of the Act/L‐Act regions quantitatively. (Certainly, different strategies or parameters, e.g., the state contrasts and the thresholds, used in activation detection will result in different Act/L‐Act regions, but this is beyond the scope of this study.) To this end, utilizing the strategies and parameters described in Barch et al.'s study (20 subjects), we used an extended dataset (30 subjects) to generate the maps and validated them by comparing them with the ones reported in Barch et al.*’*s work, in which they compared their activation maps to traditional maps in the literature to validate the HCP data quality (Barch et al., 2013).

The detection of the activation regions for the seven tasks was implemented using FSL (version 5.0.9)/FEAT (version 6.00) (Jenkinson, Beckmann, Behrens, Woolrich, & Smith, 2012). The main parameters were set as follows: At the subject level, a within‐subjects fixed‐effects analysis in FSL/FEAT was used to estimate the average effects across the runs, with a cluster‐based threshold *Z *=* *1.96 as the activation threshold and *p *=* *.05 as the Monte Carlo‐based cluster‐level correction. Then, at the group level, a mixed‐effects analysis implemented in FSL/FLAME (FMRIB's local analysis of mixed effects) (Beckmann, Jenkinson, & Smith, 2003) was used to estimate the average effects of interest, in this case, the activation maps, separately for the seven task groups, with a cluster‐based threshold *Z *=* *2.32 and *p *=* *.05. In this study, only the positive activation regions were used because, to date, the physiological interpretations of negative activations obtained from BOLD fMRI remain controversial (Bianciardi, Fukunaga, van Gelderen, de Zwart, & Duyn, 2011; Hu & Huang, 2015; Shih et al., 2009). The details of the contrasts used for each task are listed in Table S2. The comparisons from different state contrasts are also presented in the Supporting information (Table S1 and Figures S1 and S2). The activation masks for the three separate datasets (LR, RL, and averaged) are available on our website (https://github.com/nmzuo/Act-L-Act-network); the masks largely coincide across the three states.

### 2.4 Definition of network nodes

In this study, a group of regions of interest (ROIs) across the entire brain was used as brain network nodes to analyze the whole brain function (Poldrack, 2007). Specifically, we used the 264 ROIs identified by combining meta‐analyses with functional connectivity mapping, spanning the cerebral cortex, subcortical structures, and the cerebellum (Power et al., 2011). For each of the seven task states, the 264 ROIs were assigned to either Act or L‐Act regions depending on the activation map.

### 2.5 Definitions of functional connectivity network

Functional networks were then defined by assessing the functional connection strength between node pairs. Two steps were adopted to construct the connectivity matrix. First, the averaged time series for each ROI was regressed out of the mean task activity (Cole et al., 2013); then, the connection strength for each node pair was calculated using the corrcoef function in MATLAB (version 2012a, Mathworks Inc.), which was shown to yield a similar result to the psycho‐physiological interaction method (Cole, Yang, Murray, Repovs, & Anticevic, 2016); finally, a Fisher's z‐transform was performed. For the averaged dataset, the connection strength was obtained by averaging the Fisher z‐transform of the correlation coefficients for the LR and RL data from each subject (Smith et al., 2013). Because the interpretation of negative connections remains controversial (Murphy, Birn, Handwerker, Jones, & Bandettini, 2009), in this study the negative connections were set to 0 (Liu et al., 2016; Sheffield et al., 2015). Second, the connection matrix for each subject was thresholded and binarized to have different densities (5%, 10% and 15%) (Bullmore & Sporns, 2009; Rubinov & Sporns, 2010; van Wijk, Stam, & Daffertshofer, 2010) to address the effects of different connectivity densities. In this manuscript, we only present the results from using a density of 15%, with the other results presented in the Supporting information.

### 2.6 Definitions of network interaction, fractional modularity, and efficiency

Three measurements were used to evaluate the segregations and integrations of the two classes of regions, the activated regions (Act) and less‐activated regions (L‐Act), where the whole brain = Act ∪ L‐Act.

First, the interaction strength was defined to assess the integration and segregation between the Act and L‐Act regions (Liu et al., 2016; Zuo et al., 2012), as in the following Equation (1),

(1) $$I=\frac{1}{K\cdot L}\sum_{i\in \mathrm{Act},\;\;j\in L-\mathrm{Act}}A_{\mathrm{ij}},$$

where *A*_*ij*_ (*i*,* j* = 1,2…*N*,* N* is the number of nodes) is the element of the connectivity matrix *A*;* K* and *L* denote the size of the Act regions and L‐Act regions, respectively; and the term *K*·*L* is used to normalize the interaction strength.

Second, a modularity index, *Q*, was used to characterize the tendency of a network to approach modularity (Blondel, Guillaume, Lambiotte, & Lefebvre, 2008; Newman & Girvan, 2004). The modularity index is defined as,

(2) $$Q=\frac{1}{2w}\sum_{i,j\in A}\left[A_{\mathrm{ij}}-\left(\gamma^{+}\frac{w_{i}w_{j}}{2w}\right)\right]\delta \left(g_{i},g_{j}\right),$$

where $w_{i}=\sum_{j}A_{\mathrm{ij}}$ is the degrees of node *i* in *A*, the γ is the resolution parameter for the optimal modular index; *g*_*i*_ indicates the regions assignment of node *i,* and delta function δ indicates that only the fraction where the two nodes are in the same regions will be counted in the final modular index. In this study, the default resolution parameter γ = 1 was adopted when applying the generalized Louvain method (http://netwiki.amath.unc.edu/GenLouvain/GenLouvain) (Mucha, Richardson, Macon, Porter, & Onnela, 2010). This parameter setting is also the optimal one for our dataset (see Figure S6 in the Section S5 for the rationale behind the parameter settings). Moreover, to reduce the bias of random walking while optimizing the modularity, we repeated the solution 100 times, and the partition associated with the largest modularity index was selected as the final partition scheme (Vatansever et al., 2015).

To evaluate the contributions of the two classes of regions to the entire network, the fractional modularity index *Q*_f_ (Figure 1) of a region was computed using only the connections engaged in the region. Then, two kinds of *Q*_f_ could be calculated separately by including connections with (1) both nodes and (2) at least one node assigned to the one class of regions under consideration. We tested the different contributions from the two regions using a two‐way ANOVA with [Subjects * States] × [Classes of Regions], where the two columns of the design matrix were the fractional modularity indices from the Act and L‐Act regions and the rows were the 453 subjects in the seven task states. Furthermore, the 453 subjects and the seven task states were treated separately as repeated measures.

Third, the global efficiency of the Act and the L‐Act regions, as well as the entire network, was computed for both the resting states and the task states to evaluate whether the two classes of regions reconfigured to facilitate the entire network reconfiguration. The global efficiency of network A is defined as follows (Achard & Bullmore, 2007; Latora & Marchiori, 2003):

(3) $$E=\frac{1}{N\left(N-1\right)}\sum_{i\neq j,\in A}\frac{1}{d_{\mathrm{ij}}},$$

where *d*_*ij*_ denotes the shortest path between the pair of nodes (*i*,* j*), which was calculated using Dijkstra's algorithm (Latora & Marchiori, 2003). When we focused on the efficiency of a local area, only the connections inside this area, such as the Act regions or the L‐Act regions, were counted. The network efficiency of a node is based on the shortest path needed to assess the communication capability of the node with other nodes across the brain. Then, the global efficiency is the mean value of all the nodal efficiencies. In this study, this measure was used to measure the functional connectional efficiency of both the global network and the Act/L‐Act regions quantitatively. The network efficiency was computed using open‐access software developed by our group (https://www.nitrc.org/projects/brat).

### 2.7 Measurements of the network flexibility from resting to task states

The previous sections have described how we investigated whether the network architecture of the L‐Act regions, concomitant to the active ones, also reconfigures when the brain changes from the resting state to task states. For a closer look at which nodes or subnetworks make the most contributions to the network dynamics, the participant coefficient (PC) (Guimera & Nunes Amaral, 2005) was utilized to measure the participant intensity of each functional node or subnetwork during mental state changes. The flexibility was defined as *F*_*k*_ = PC_*ik*_−PC_*jk*_, where i and j indicate two mental states, that is, a task and the resting state, for a specific node k. The subnetworks defined by Power et al. (2011) were used, and the individual subnetwork flexibility was examined by averaging the flexibility of the nodes composing the subnetwork. Ten of 13 subnetworks were included in our study, because the other three subnetworks do not have specific and determined functional roles (Cole et al., 2013; Vatansever et al., 2015).

## 3 RESULTS

### 3.1 Global network efficiency comparisons and modular changes

**Figure 2:**
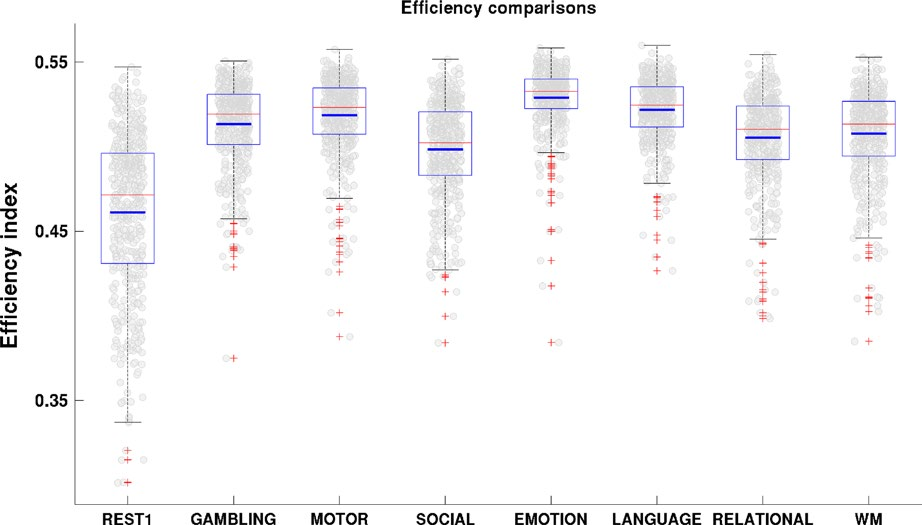
The efficiency indices for the whole brain in different mental states, including the resting state and the seven tasks, that is, gambling, motor, social cognition, emotion processing, language, relational processing, and working memory tasks. The red (long) and blue (short) horizontal lines in each box, respectively, denote the median and mean efficiency indexes across all of the 453 subjects. For a closer look at the distribution of the indices for the 453 subjects, the scatter plot of each subject's index is overlaid as the background of each plot

The comparison‐based global efficiency index showed that the brain networks had much higher efficiency in the task states than in the resting states. Figure 2 shows plots of the efficiency indices for the whole brain, including those for the resting state and each of the seven task states. From these plots, the efficiency indices for the seven task states can clearly be seen to be larger than those for the resting state. The results for the paired *t* test were all significant (*p *<* *1 × 10^−36^) for the seven task‐resting pairs, and their *p*‐values survived false discovery ratio (FDR) correction for multiple comparisons using the mafdr function in MATLAB (corrected *p*‐values <1 × 10^−35^). Furthermore, because the distributions for the modularity indices for the eight mental states did not strictly follow a Gaussian normal distribution (they did not pass the Kolmogorov–Smirnov test by kstest in MATLAB), a complementary paired permutation test was used with 10,000 permutations, and the results consistently showed *P *=* *0. The results with other connectivity density thresholds, 5% and 10%, and three separate dataset are presented in Figure S3.

**Figure 3:**
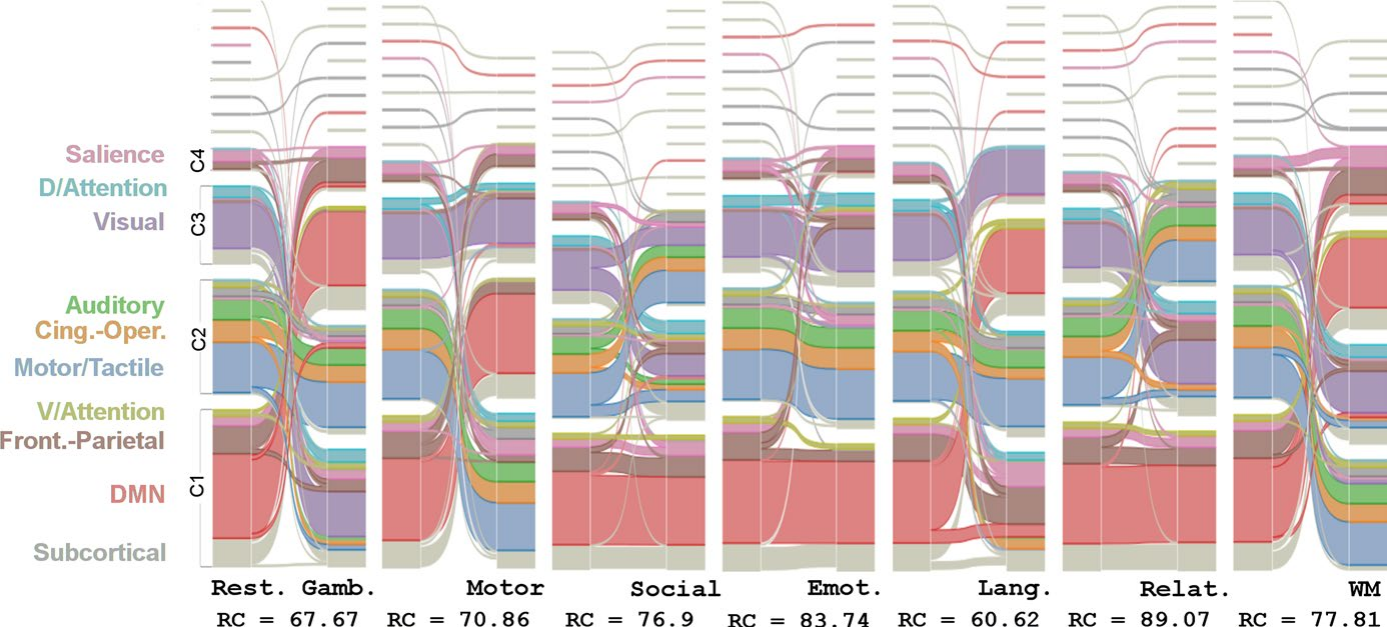
Global changes by alluvial diagram (Rosvall & Bergstrom, 2010) of the node assignments to the two different regions (Act and L‐Act) between the baseline (resting state) and the seven task states. The region partitions for each state are the average partitions obtained by maximizing the similarity across the 453 participants separately for each state (Bassett et al., 2013). Here, only large partitions are labeled by *C*_*i*_ (*i* = 1, 2, …), and different regions are separated by white gaps in the horizontal direction. The seven panels indicate the seven resting‐task pairs where the left one is for the resting and the right one is for the task. The last row indicates the similarity, measured by the *z*‐score of the random coefficient (RC) (Traud et al., 2011) between the partitions from the resting state and the task state

Figure 3 illustrates the global dynamic changes by an alluvial diagram (Rosvall & Bergstrom, 2010) for node assignments to either the baseline (resting state) or the seven task state communities. The partitions between the communities for each state, including both the baseline (resting state) and the seven task states in Figure 3, were the representative partitions across the 453 partitions based on statistical testing in comparison with a null model (Bassett et al., 2013). The node flow diagrams show the intensive reconfigurations of the networks when the brain switched from the resting state to the seven task states. In Figure 3, the last row indicates the similarity measured by the z‐score of the random coefficient (RC) (Traud, Kelsic, Mucha, & Porter, 2011) between the partitions from the resting state and from the task state. For a description of the connectivity flow obtained by grouping the nodes into Act and L‐Act regions, see Section S6 where the connectivity matrices (Figure S7) that illustrate the connectivity reconfigurations when the brain changed state from the resting to a task state are shown.

### 3.2 Changes in the interactions between the Act and L‐Act regions

**Figure 4:**
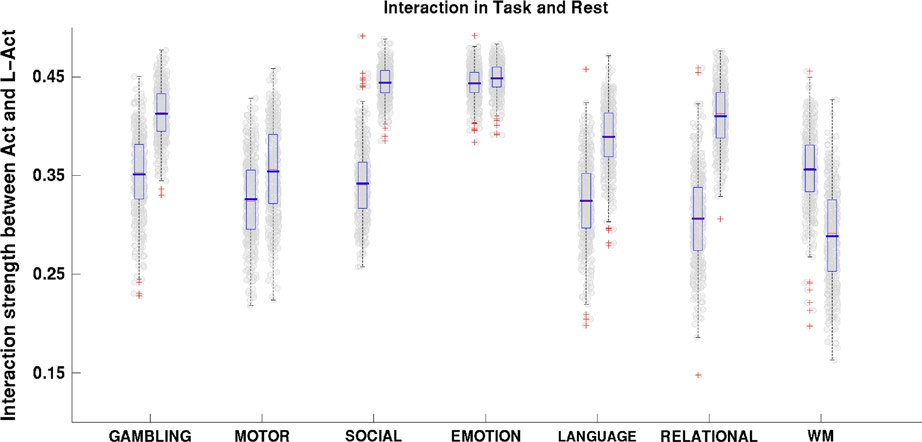
Comparisons of the strength of the interaction between the Act and L‐Act regions in the resting and task states. Within each column pair, the left shows the interaction between the two regions in the task state and the right shows the resting state

We further found that the interactions between the Act and L‐Act regions changed significantly along with the global change in efficiency. Figure 4 shows the comparisons of the strength of the interactions between the Act and L‐Act regions in the resting and task states. In each of the seven pairs of columns, the left presents the interaction strength between the two classes of regions in the task state and the right presents that in the resting state. The *p*‐values for the paired *t* test for the seven task‐resting pairs were (*df* = 452): gambling: *p *=* *7.1 × 10^−108^, *t *=* *−29.6; motor: *p *=* *1.64 × 10^−22^, *t *=* *−10.3; social cognition: *p *=* *4.27 × 10^−215^, *t *=* *−59.21; emotion processing: *p *=* *2.16 × 10^−7^, *t *=* *−5.27; language: *p *=* *6.46 × 10^−102^, *t *=* *−28.3; relational processing: *p *=* *6.16 × 10^−165^, *t *=* *−43.85; and WM: *p *=* *9.28 × 10^−94^, *t *=* *26.43. These *p*‐values also readily survived FDR correction. The comparisons show that the interactions between the Act and L‐Act regions were significantly different in the task states compared with their interactions in the resting states. Specifically, the interactions weakened in six of the task states but strengthened in the WM task.

**Figure 5:**
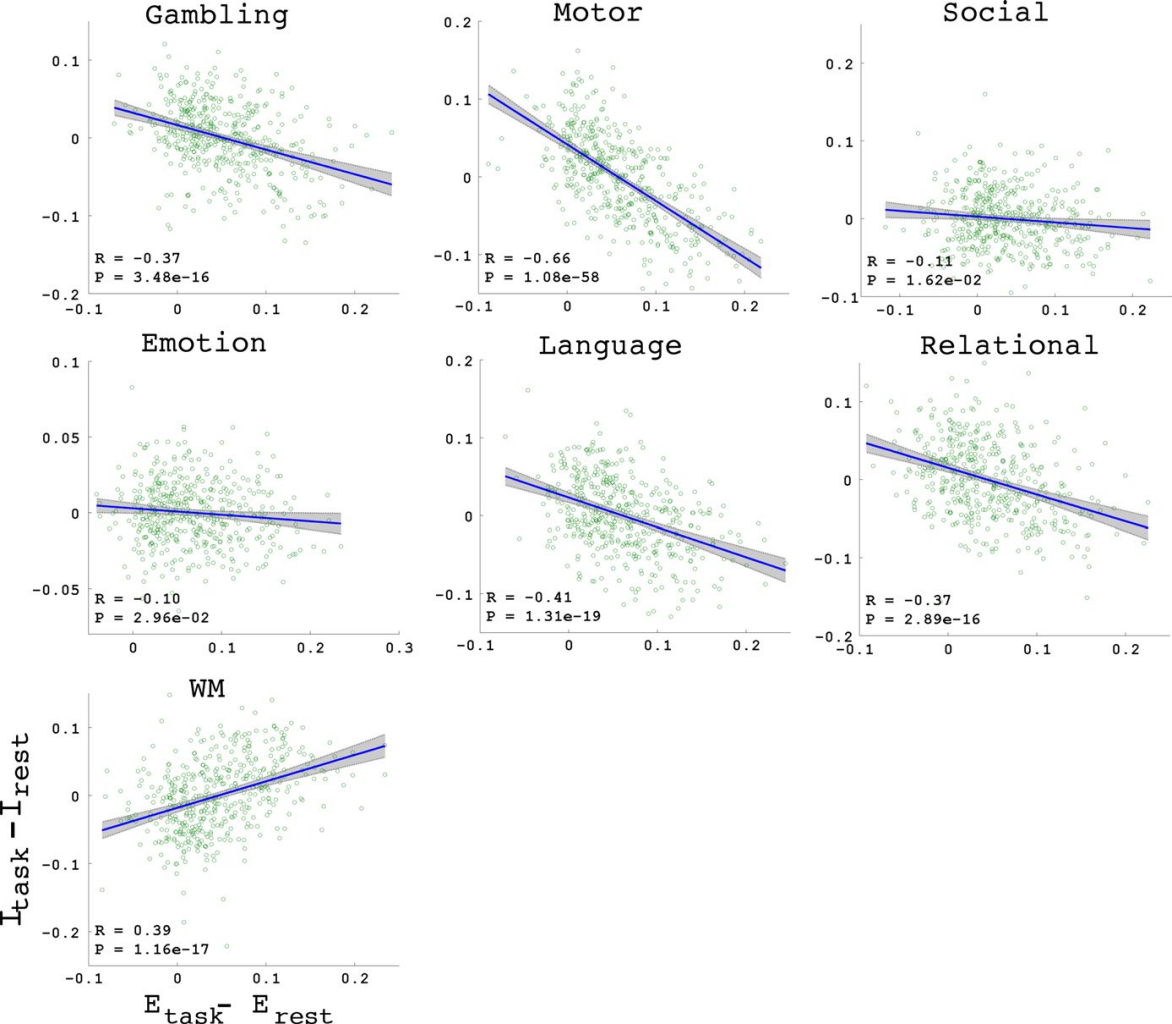
Correlations between the changes in the interactions between the two regions and the changes in the global efficiency. The individual correlation strength *R* and significance level *p*‐value appear in each panel

We also found that the changes in the interaction strengths correlated with the global efficiency changes when the brain switched mental states. Figure 5 shows the correlation test results for the 7 tasks, with the sex and age of each subject added as confounding factors. These results consistently demonstrated that changes in the interactions between the Act and the L‐Act regions were significantly correlated with changes in the efficiency after FDR correction. The results with other connectivity density thresholds, 5% and 10%, and three separate datasets are presented in Table S3 and Figure S4.

### 3.3 Fractional modularity analysis

Using a two‐way ANOVA with [Subjects * States] × [Classes of Regions], we found that, for the entire network, the fractional modularity contributions of the L‐Act regions were not statistically significantly different from those of the Act regions (the main effect *p *>* *.11). In Table 2, the results are classified by the node‐pair assignment strategy (e.g., whether the connected nodes were both in Act or both in L‐Act or one of each pair of nodes was in Act and the other in L‐Act), and the fractional modularity contributions were normalized by two different factors, the size of each class of connected regions or the number of all possible connectivities between these two classes of regions. The last row of Table 2 separately denotes the main effects for all the cases above. These results consistently showed that the modularity contributions from the Act and L‐Act regions did not differ significantly in any of the seven task states. The interaction effects were insignificant (*p *>* *.86) between the factors of the subject (or task) and the two classes of regions. The results with other connectivity density thresholds, 5% and 10%, and three separate datasets are presented in Table S5.

**Table 2:** Comparisons of the fractional modularity *Q*_f_ contributed separately by the Act and L‐Act regions, classified by node‐pair assignment, normalization scheme, and repeated measures. The reps designation indicates the number of repeated measures. Two methods were considered and repeated according to the number of subjects and according to the number of tasks. The last row indicates the main effects from the two‐way ANOVA and is the difference in the two columns composed by the contributions separately from the Act and L‐Act regions in the ANOVA design matrix. The interaction effects between the subjects (tasks) and the Act/L‐Act difference were not significant (*p* > .86)

Normalized by *C*(*k*,2)^a^				Normalized by Act/L‐Act size			
Both nodes (*i,j*) are in the examined regions		At least one node of (*i,j*) is in the examined regions		Both nodes (*i,j*) are in the examined regions		At least one node of (*i,j*) is in the examined regions	
reps = 453	reps = 7	reps = 453	reps = 7	reps = 453	reps = 7	reps = 453	reps = 7
*F *= 2.24*p *= .13	*F *= 2.24*p *= .13	*F *= 0*p *= .93	*F *= 0*p *= .93	*F *= 2.52*p *= .11	*F *= 2.51*p *= .11	*F *= 0.95*p *= .33	*F *= 0.95*p *= .33

a The *C*(*k*, 2) means the possible connections between *k* nodes, and the *k* nodes are constrained by the rule in the second row of the table.

### 3.4 Network changes within the Act and L‐Act regions

**Figure 6:**
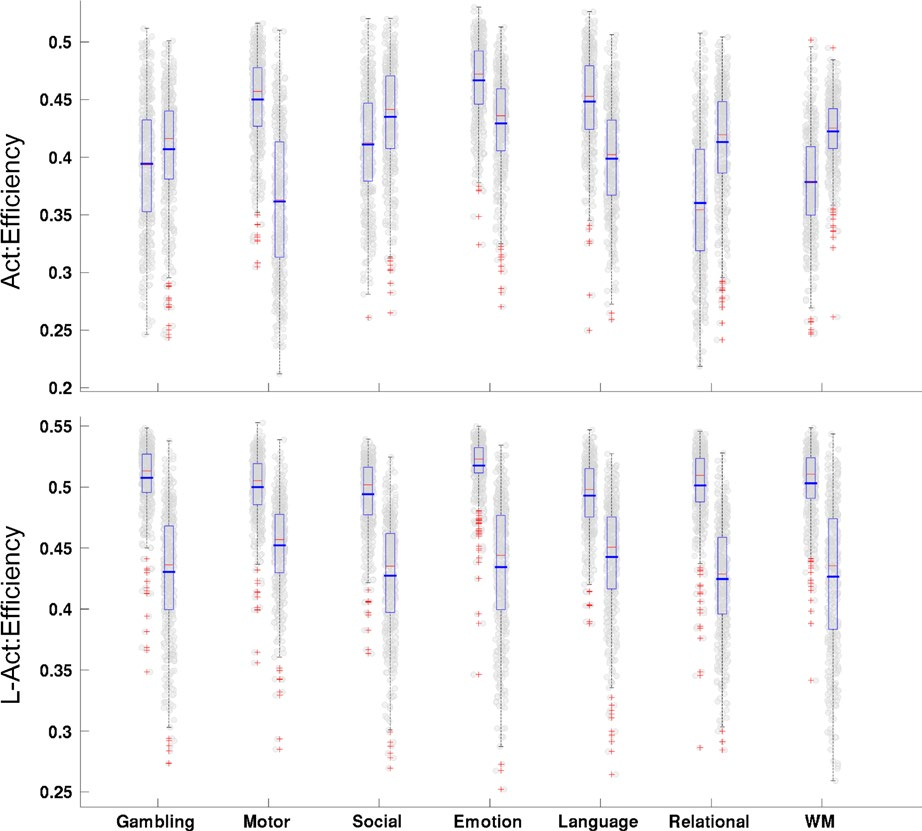
Comparisons of efficiency between the task states and resting state for Act (top panel)/L‐Act (bottom panel) regions. The 7 column pairs in each panel indicate each of the seven task states. The left one of each column pair shows the task state, and the right shows the resting state. The results for the Act regions do not show consistent increase trends from the resting state to the task state although there was a statistically significant difference between the resting and task states

Using the areal efficiency (the network efficiency in either Act or L‐Act) to characterize the changes in these two classes of regions, we found that both the Act and the L‐Act regions had significantly reorganized internal network architectures (*p *<* *1.0 × 10^−4^ after FDR correction) when they changed from the resting to the seven task states. In particular, the efficiency in the L‐Act regions consistently increased (see Figure 6).

**Figure 7:**
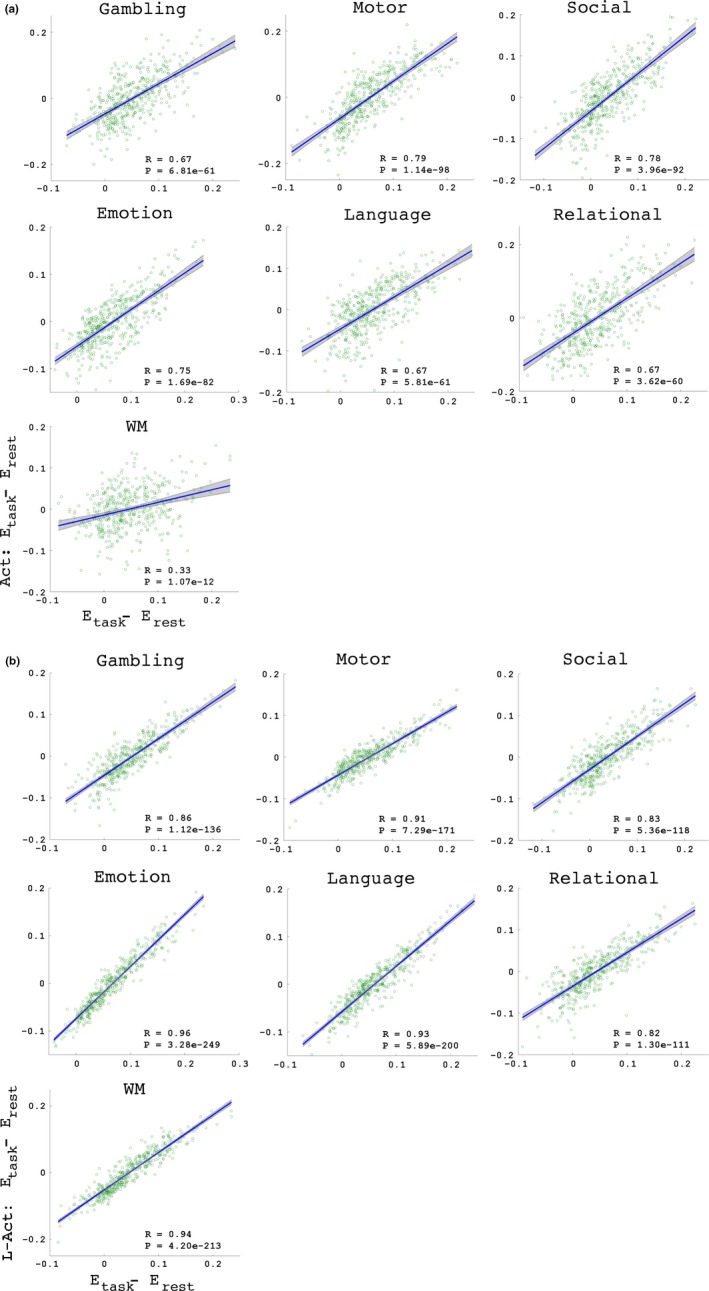
Correlations between the changes in the efficiency indices between the two classes of regions and the changes in the global efficiency. The individual correlation strength *R* and significance level *p*‐value are presented in each panel. Panels a and b indicate the Act and L‐Act regions, respectively

Figure 7 shows the correlation test results between the changes in the efficiency of the Act (7a) and L‐Act (7b) network when switching from the resting state to the seven task states. These results consistently demonstrated that the changes in the efficiency in both the Act and L‐Act regions were significantly correlated with the changes in the global efficiency indices. The results with other connectivity density thresholds, 5% and 10%, and three separate datasets are presented in Table S4 and Figure S5.

### 3.5 Flexibilities of the networks during the changes from the resting to the task states

**Figure 8:**
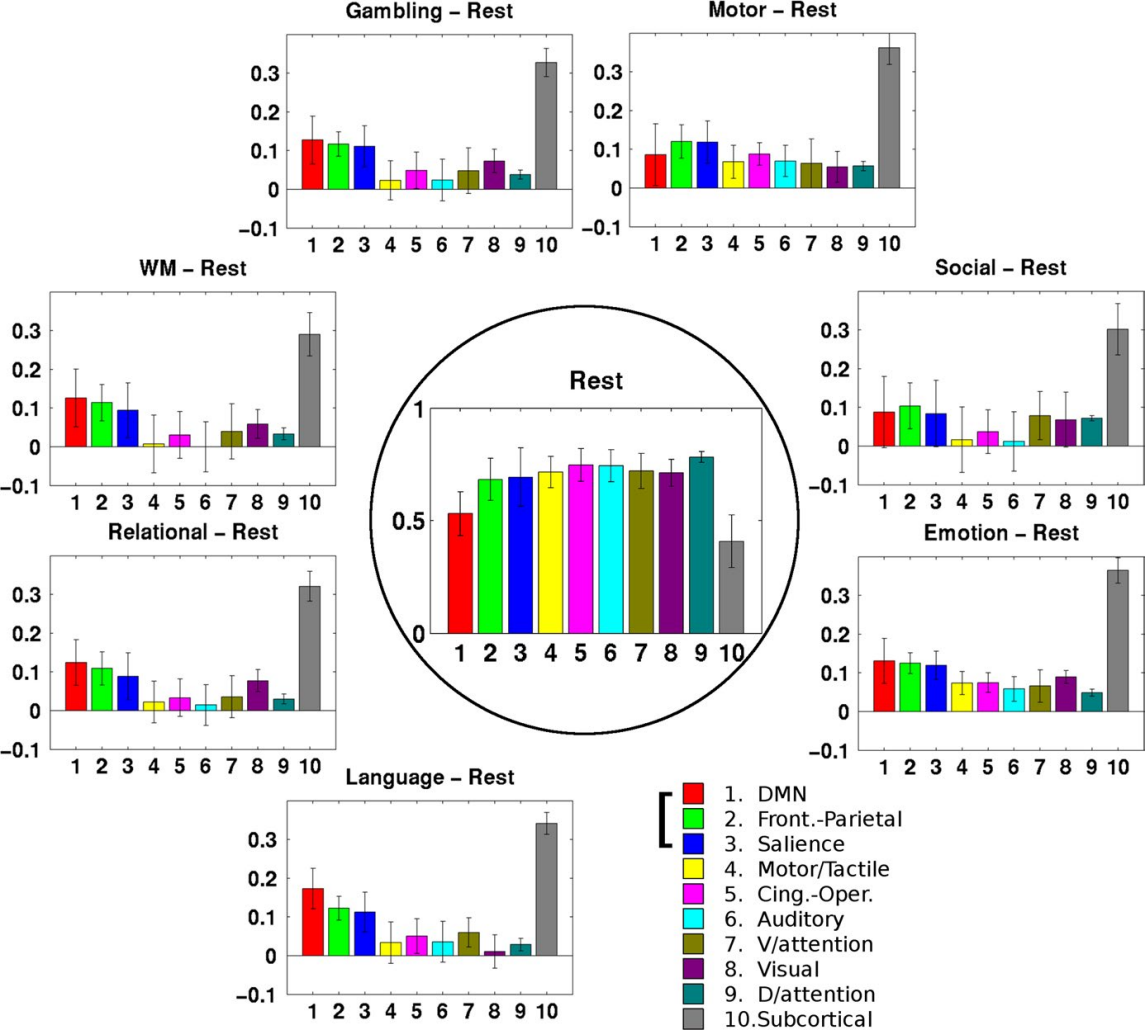
Comparisons of the participant coefficients (the central one for the resting state) and the flexibilities (the surrounding ones for the seven resting‐tasks pairs) for the subnetworks. As the right‐bottom panel shows, each colored bar indicates the specific subnetworks. Except for the subcortical network, the DMN, FPN, and SN networks had the greatest flexibility (*p* < 1.0 × 10^−28^ after FDR correction when comparing their mean flexibility with the mean flexibility of the other six networks across the 453 subjects)

The flexibility metric for the subnetworks showed that, across the seven resting‐task state pairs, the default mode network (DMN), frontoparietal network (FPN) and salience network (SN) consistently had the greatest flexibility among the 10 subnetworks. In Figure 8, the central panel shows the PCs of the 10 subnetworks in the resting state and the surrounding panels show the flexibilities of each subnetwork from the resting state to the seven task states. With the exception of the subcortical network, the DMN, FPN, and SN networks had the greatest flexibility (*p *<* *1.0 × 10^−28^ after FDR correction when comparing their mean flexibility with the mean flexibility of the other six subnetworks across the 453 subjects). The specific statistical *p*‐values are 2.6 × 10^−74^ (gambling), 8.68 × 10^−28^ (motor), 2.17 × 10^−30^ (social cognition), 1.72 × 10^−45^ (emotion processing), 1.55 × 10^−110^ (language), 6.72 × 10^−71^ (relational processing) and 1.97 × 10^−88^ (WM). The high flexibility of the subcortical network is logical because the subcortical network consists of many heterogeneous functional nodes globally regulating the other subnetworks (Hibar et al., 2015).

## 4 DISCUSSION

The goal of this study was to investigate the roles of both the activated regions (Act) and the less‐activated regions (L‐Act) of the brain during the execution of tasks, where Act refers to the brain regions “significantly activated” in the literature in a task‐related fMRI analysis and L‐Act refers to the part of the brain that is complementary to Act (Yamashita et al., 2015). Our research disclosed the following findings of the brain network about the task states in comparison with the resting states: (1) We found that the interaction strength between the Act and L‐Act regions changed significantly during the brain state changes, and this change was associated with a change in the brain network efficiency. (2) We found that the L‐Act regions made contributions, quantified by the proposed fractional modularity index, to the global modularity change that were comparable to the contributions of the Act regions. (3) We found that the default mode network (DMN), frontoparietal network (FPN), and salience network (SN) consistently showed greater flexibility than the other subnetworks when the brain was changing from the resting state to the seven task states. These findings indicate quantitatively that executing a task will recruit multiple, if not brain‐wide, subnetworks, rather than only activating task‐specific regions. All these findings have been repeated using three datasets (LR, RL, and the averaged) with different network density thresholds (5%, 10%, and 15%). (See more results in the Supporting information.) Across the three thresholds, the main results showed great consistency, including in comparisons of the global efficiency, in the correlations between the changes in interaction strengths, in the changes in efficiency, and in the connection changes in fractional modularity. The changes in the interactions between the activated and less‐activated regions from the resting to the tasks also showed considerable consistency, except that the gap in the comparison between the resting and EMOTION task was reduced when the threshold changed from 15% to 5%, but it still showed increased segregation from the resting to the task state. A few brain‐wide studies exploring the common patterns in different mental states have been reported (Betti et al., 2013; Bolt, Nomi, Rubinov, & Uddin, 2017; Cole, Bassett et al., 2014; Krienen, Yeo, & Buckner, 2014), but no one, to our knowledge, has quantitatively examined the network reorganization by comparing the Act and L‐Act regions. In this study, we examined the reconfigurations of the Act and L‐Act regions identified by a traditional activation detection strategy, from the perspective of functional connectivity and network analysis.

The resting state of the brain has a great similarity with its task‐processing state, in both its energy metabolism (Raichle et al., 2001) and its functional network architecture (Cole, Bassett et al., 2014). Such similarities also have a neural basis, which can be characterized by diffusion MRI (Hermundstad et al., 2013). Therefore, it is quite reasonable to think that the properties of the resting state network (not the task state) may be able to identify specific individuals (Hearne, Mattingley, & Cocchi, 2016) and predict their cognitive performance (Tavor et al., 2016). On the other hand, although the changes evoked by a task stimulus are subtle (Betti et al., 2013; Cole, Bassett et al., 2014; Gratton, Laumann, Gordon, Adeyemo, & Petersen, 2016), the reconfiguration of the network architecture from the resting state to the task state has received increasing attention, and some global network measures, such as efficiency and modularity, rather than just the metrics constrained in the Act regions, have been applied to predict individual performance (Hearne et al., 2016), intelligence (Schultz & Cole, 2016), and even the aging process (Gallen et al., 2016). Therefore, the current study bolsters our understanding of the underlying principles of how Act and L‐Act behave and interact during global network reconfigurations.

### 4.1 Segregation of and integration between the Act and L‐Act regions

Two types of findings can be drawn from the results: (1) With the exception of the working memory (WM) task, the other six tasks consistently resulted in reduced interactions between the Act and L‐Act regions. This may indicate that the six tasks specifically recruit the Act regions while the L‐Act regions act in assistant roles. However, WM is a highly cognitively demanding task that recruits an enormous number of functional regions across the brain (Constantinidis & Klingberg, 2016; Eriksson, Vogel, Lansner, Bergstrom, & Nyberg, 2015; Ullman, Almeida, & Klingberg, 2014). (2) Although all the L‐Act regions for the seven tasks consistently showed increasing trends in network efficiency when the brain transitioned from the resting state to a task state (Figure 6), they did not show unified relationships between the global efficiency changes and the Act‐L‐Act interaction changes (Figure 5). Specifically, for the six tasks other than WM, more segregations between the Act/L‐Act regions correlated with a higher global network efficiency, but for the WM task, apparently due to the global recruiting of the functional regions, fewer segregations correlated with higher global network efficiency (Godwin, Barry, & Marois, 2015). This last finding may be due to an increased, long‐distance functional synchrony across the Act and L‐Act regions when exposed to awareness‐demanding tasks (Giessing, Thiel, Alexander‐Bloch, Patel, & Bullmore, 2013; Godwin et al., 2015). Segregation and integration between functional regions are critical for enabling the brain to optimize its computational resources while controlling the wiring cost (Bullmore & Sporns, 2012; Petersen & Sporns, 2015; Sporns, 2013).

In this study, when we computed the efficiency of an areal network, for example, the Act or the L‐Act, only the internal connections were counted. According to graph theory, the increased global efficiency was not necessarily associated with increased areal efficiency. Therefore, our results collectively indicated that changes in the interactions between the Act and L‐Act regions may have been a source of the changes in the modular trend and then in the increased efficiency of the global brain network. Being able to discriminate between Act and L‐Act regions in activation detection studies does not necessarily indicate that there is no strong functional connectivity between them since the strategies of identifying activations and connections characterize different facets of the brain functioning (Bassett et al., 2015; Siebenhuhner, Weiss, Coppola, Weinberger, & Bassett, 2013).

### 4.2 The Act and L‐Act regions reconfigured comparably to enable the global network changes

Judging from our study, the brain changes globally for a single task, which is the main reason why graph theory, which emphasizes that different functional regions serve in different roles to direct the engagement of the others (Bressler & Menon, 2010), have been borrowed to address the way in which the brain works (Sporns, 2011). Joint efforts between functional regions are universally found in the brain, especially when it is exposed to external stimuli. For example, an interaction pattern was found in an excitatory‐inhibitory counterpart between the salience network (main actor) and the central executive network when the brain processes unexpected events in the environment (Palaniyappan, Simmonite, White, Liddle, & Liddle, 2013). In a whole brain network study that was similar to ours, Bassett et al. (2015) found that after visual‐motor dual‐task training, the nonvisual‐motor regions of the brain acted as a potential driver promoting the motor‐visual integration needed to perform the acts. In the literature, the active regions are often regarded as the actor, that is, that they are in charge of executing the tasks (Eickhoff, Bzdok, Laird, Kurth, & Fox, 2012; Wager, Lindquist, & Kaplan, 2007). Meanwhile, as shown by our experimental results from the perspective of the brain network, the L‐Act regions made comparable contributions and changed in ways that may possibly enable them to act as directors behind the scenes organizing the computational resources to facilitate the task execution. A similar result was presented by Yamashita and colleagues when predicting the learning plateau using the connectivity between the Act and L‐Act regions (2015). Collectively, these results are well in line with the “driver network” hypothesis proposed in Bassett et al.'s work (2015). Thus, an actor‐director model may provide a new dimension for characterizing the collaborations between the traditional Act and L‐Act regions.

### 4.3 A triple‐network model dominating the flexibility during the state changes of the brain

The three subnetworks that we found to be the most important to our study, the DMN, the FPN, and the SN, have been investigated extensively in recent years. A triple network comprising these has been proposed as a unifying model that globally regulates various brain functions (Menon, 2011), including attention and inhibitory control, execution capability. This regulation process may be implemented by integrating the different roles of these three networks (Chen et al., 2013; Sridharan, Levitin, & Menon, 2008). The FPN is responsible for coordinating load‐specific cognitive resources (Cai et al., 2016), for example, for exerting inhibitory control on the DMN activity when excited by an external stimulus (Chen et al., 2013; Sherman et al., 2014). The appropriate assignment of that regulation seems to be performed by the SN (Cai et al., 2016; Sheffield et al., 2015). This triple‐network configuration is strengthened during developmental maturation (Sherman et al., 2014; Supekar & Menon, 2012). In this study, we quantitatively supported the dominance of the flexibility of these three networks when they were engaged in different task executions (seven tasks in total), including visual/auditory/motor tasks, memory and retrieval, attention, and rewards. Noteworthily, these three subnetworks are not constrained to certain Act or L‐Act regions. Because of the pivotal roles of these three networks, aberrant engagement and disengagement in them can cause various psychiatric and neurological disorders (Cole, Repovs, & Anticevic, 2014; Menon, 2011), including schizophrenia (Sheffield et al., 2015), anxiety disorders (Sylvester et al., 2012), obsessive‐compulsive disorder (Stern, Fitzgerald, Welsh, Abelson, & Taylor, 2012), borderline personality disorder (Doll et al., 2013), and psychopathy (Philippi et al., 2015).

## 5 CONCLUSIONS

The activation region detected from functional MRI data is generally recognized as the main actor in performing a dedicated task. However, what the L‐Act regions are doing at the same time remains largely unknown. This study quantitatively demonstrated that both the Act and the L‐Act regions underwent segregation and integration in ways that resulted in a reconfiguration of the global network. Furthermore, both types of regions reorganized comparably to support the modular changes in the global brain network. More importantly, we discovered that the default mode network, the frontoparietal network, and the salience network consistently had the greatest flexibility compared to other subnetworks across the seven different tasks. These findings quantitatively signify that executing a task recruits multiple, if not brain‐wide, subnetworks, rather than only activating task‐specific regions. The clarification of the relationship between the Act and L‐Act regions and their roles in the global network reconfigurations may provide a new perspective for understanding the changes in the brain network when exposed to cognitively demanding tasks and establishes the necessity of using network theory to investigate brain functions in imaging cognitive neuroscience.

## CONFLICT OF INTERESTS

None declared.

## AUTHOR CONTRIBUTIONS

N.Z. and T.J. designed research; N.Z., S.Y., and Y.L. performed research; N.Z. and J.L. analyzed results; and N.Z. documented results and wrote the paper.

## DATA STATEMENT

Data were provided by the HCP, WU‐Minn Consortium (Principal Investigators: David Van Essen and Kamil Ugurbil; 1U54MH091657) funded by the 16 NIH Institutes and Centers that support the NIH Blueprint for Neuroscience Research, and by the McDonnell Center for Systems Neuroscience at Washington University.
