# Supplementary material for: Both activated and less‐activated regions identified by functional MRI reconfigure to support task executions
Source: Brain Behav. 2017 Dec 20;8(1):e00893. doi: 10.1002/brb3.893 (PMC5853621; doi:10.1002/brb3.893)
Supplement: Supplementary file 1 [file BRB3-8-e00893-s001.pdf]

# Both Activated and Less-Activated Regions Identified by Functional MRI Reconfigure to Support Task Executions

Nianming Zuo, Zhengyi Yang, Yong Liu, Jin Li and Tianzi Jiang

Supplementary Materials

This document is presented as a supplement for adding details of our methods and validating the results in the manuscript using other datasets (LR/RL phase encodings and the averaged networks (abbr. AVG)) and different thresholds (5%, 10% and 15% densities) in constructing connectivity networks. The considerations on the choices of task contrasts in activation detection will be discussed as well. Finally, we will provide the details of our implementation, including the subject list for activation detection and the results of the activation detection as NIFTI files. (***All the source code in Matlab in this study,*** https://github.com/nmzuo/Act-L-Act-network .)

Section S1: Details of data preprocessing and activation detection

The HCP dataset has already been partially applied in our previous work *(Zuo et al., 2016)*. For the purpose of completeness, we repeat the main descriptions and preprocessing steps here. The dataset was collected on a 3T MRI Skyra scanner (Siemens, Germany) using a standard 32 channel head coil. The magnetic field produced by the coil was modeled to provide a customized distortion correction. The primary scanning parameters were as follows: repetition time (TR), 720 ms; echo time (TE), 33.1 ms; flip angle, 52°; field of view, 208 × 180 mm; slice thickness, 2.0 mm; and voxel size, 2.0 mm isotropic cube *(Van Essen et al., 2013)*.

The HCP data was already preprocessed, well aligned and registered to the Montreal Neurological Institute (MNI) 2 mm standard space. The main preprocessing steps taken include *(Glasser et al., 2013)*: (i) gradient nonlinearity distortion; (ii) 6 degrees of freedom (DOF) FSL/FLIRT-based motion correction; (iii) FSL/topup-based distortion correction; (iv) registration to a T1 space image; and (v) FSL/FNIRT-based registration to MNI 2 mm space. After receiving the data, we further band-pass filtered the data at 0.009–0.08 Hz to reduce low-frequency drift and high-frequency noise *(Vatansever et al., 2015)*. The mean signal of the white matter and cerebrospinal fluid (CSF), and the movement parameters and its derivatives (in the Movement_parameters.txt file in HCP S500 release) were regressed out as confounding factors. Since we used a series of ROIs in this study to sample the gray matter of the entire brain (described below), a smoothing step was not applied here. In addition, global signal regression was not conducted because its use is controversial and there is no consensus about its physiological interpretation *(Bassett et al., 2015; Mayhew et al., 2016)*.

The detection of the activation regions for the 7 tasks was implemented by FSL (version 5.0.9)/FEAT (version 6.00) *(Jenkinson et al., 2012)* using parameter settings that were extremely similar to those in Barch *et al.*'s report *(2013)*, the key steps of which are described here. Both the fMRI time series and the GLM were temporally filtered with a Gaussian-weighted linear highpass filter with a cutoff of 200 s. The volume data was spatially smoothed using a 3D Gaussian kernel with FWHM = 4 mm. Since the preprocessed data from the HCP had already been skull-stripped and realigned to MNI 2 mm space, these operations, including motion correction and brain tissue extraction in the FSL/FEAT GUI, were not repeated. The FILM prewhitening step was chosen to increase the validity and efficiency of the statistics *(Woolrich et al., 2001)*. Three items, the mean white matter signal, the cerebrospinal fluid signal, and the 12-item head movement parameters (from the S500 dataset), were set as confounding variables.

The subsequent statistical analysis was conducted in two steps. At the subject level, a within-subjects fixed-effects analysis in FSL/FEAT was used to estimate the average effects across the runs, with a cluster based threshold Z = 1.96 as the activation threshold and P = 0.05 as the Monte Carlo-based cluster-level correction. Then at the group level, a mixed-effects analysis implemented in FSL/FLAME (FMRIB's Local Analysis of Mixed Effects) *(Beckmann et al., 2003)* was used to estimate the average effects of interest separately for the 7 task groups, with a cluster-based threshold Z = 2.32 and P = 0.05. In this study only the positive activation regions were used since, to date, the physiological interpretations of negative activations obtained from BOLD fMRI remain controversial *(Bianciardi et al., 2011; Hu and Huang, 2015; Shih et al., 2009)*.

Section S2: Act and nonAct definitions

As described in the Methods section in the manuscript, for the strategy discriminating Act/nonAct regions, we seek for a balance between the specificity and generalizations of functional activations. This means when try to constrain the regions for a specific task, we should also guarantee that the size of regions are not too small and able to cover a sufficient number of ROIs from the Power264 ROI list *(Power et al., 2011)* for network modularity evaluation.

The task contrasts used for each task were to detect task-specific functional regions and remove any confounding activations. For example, in the WM task, the subjects were cued by pictures on the screen to make inferences and decisions. The activation maps of [2back – 0back] and [2back – baseline] were significantly different. The choice of [2back - baseline] resulted in many areas of the primary cortex being activated, such as the visual cortex (see Fig. S5 in the Supplemental Materials). In this study, however, we were interested in higher cognitive functions, including planning and problem-solving *(Cohen et al., 1997)*, so we adopted the contrast of [2back – 0back] instead of [2back - baseline]. In addition, we tried to avoid generating activation maps which were too small, because too few ROIs would lead to unstable network construction and analysis. For example, for the gambling task or the motor task, we chose alternative contrasts rather than contrasts that only activate a very few specific areas. The details of the contrast used for each task are listed in Table 1. The activation masks for the three separate datasets (LR, RL and the averaged) are available on our website (https://github.com/nmzuo/Act-L-Act-network) and the masks largely coincide across the three states.

Here in Table S1 we list all the contrasts for each task state and the descriptions explaining the choice in the manuscript. About the principles how these contrast were designed and potential activated regions please refer to the Human Connectome Project (HCP) website, http://www.humanconnectome.org/documentation/Q1/task-fMRI-protocol-details.html and http://www.humanconnectome.org/documentation/phase1-paradigms-fMRI.html. All the activation maps can be downloaded (see Appendix B). Table S2 listed the contrast used in the manuscript.

Table S1. The contrasts for each task used in the manuscript are based on a series of pre-studies. The name "cope" is in line with the routine of the FSL/FEAT, which means contrast of parameter estimates *(Jenkinson et al., 2012)*

Task	All contrasts we have conducted	Reasons for the choices in the manuscript
Gambling	cope1: reward vs. fixation;<br>cope2: reward vs. neut;<br>cope3: reward vs. punish;	Use cope1. The result from cope2 is nearly empty and from cope3 contains too small areas (shown in Fig. S1).
Motor	cope1-5: (Left-Hand, Right-Hand, Left-Foot, Right-Foot, Tongue) vs. Fixation	Combined 1-5. Motor task activates many isolated regions in the individual Left-Hand, Right-Hand, Left-Foot, Right-Foot and Tongue sessions, so we combined all the individual small regions into an integral one.
Social	cope1: social vs. baseline;<br>cope2: social vs. rand;	Use cope1. Cope2 has very similar area as Cope1.
Emotion	cope1: Fear vs. neutral	Use cope1. No fixation and have only one cope.
Language	cope1: Story vs. Math	Use cope1. No fixation and have only one cope. Here we do not discriminate short sessions, including present, question and response.
Relational	cope1: relational vs. fixation;<br>cope2: relational vs. match;	Use cope1. The activation regions from the cope2 are too small (shown in Fig. S1).
WM	cope1: 2bk-0bk;<br>cope2: 2bk-baseline;<br>(combine (body, face, place, tool) as a whole)	Use cope1. By contrast to the cope2, the results from cope1 denote the core regions which would be called by more difficult cognitive tasks. Actually, Contrasting Fig. S1 to Fig. 1, they share most areas since they perform very similar tasks except for the difficulty level.

Table S2. Contrast designs for the 7 task paradigms for generating activation map in the manuscript.

Task	Emotion	Gambling	Language	Motor	Relational processing	Social cognition	Working Memory

Contrast	Fear vs. Neutral	Reward vs. Fixation	Story vs. Math	All 5 Tasks-Fixation	Relational vs. Fixation	Social vs. baseline	2back vs. 0back

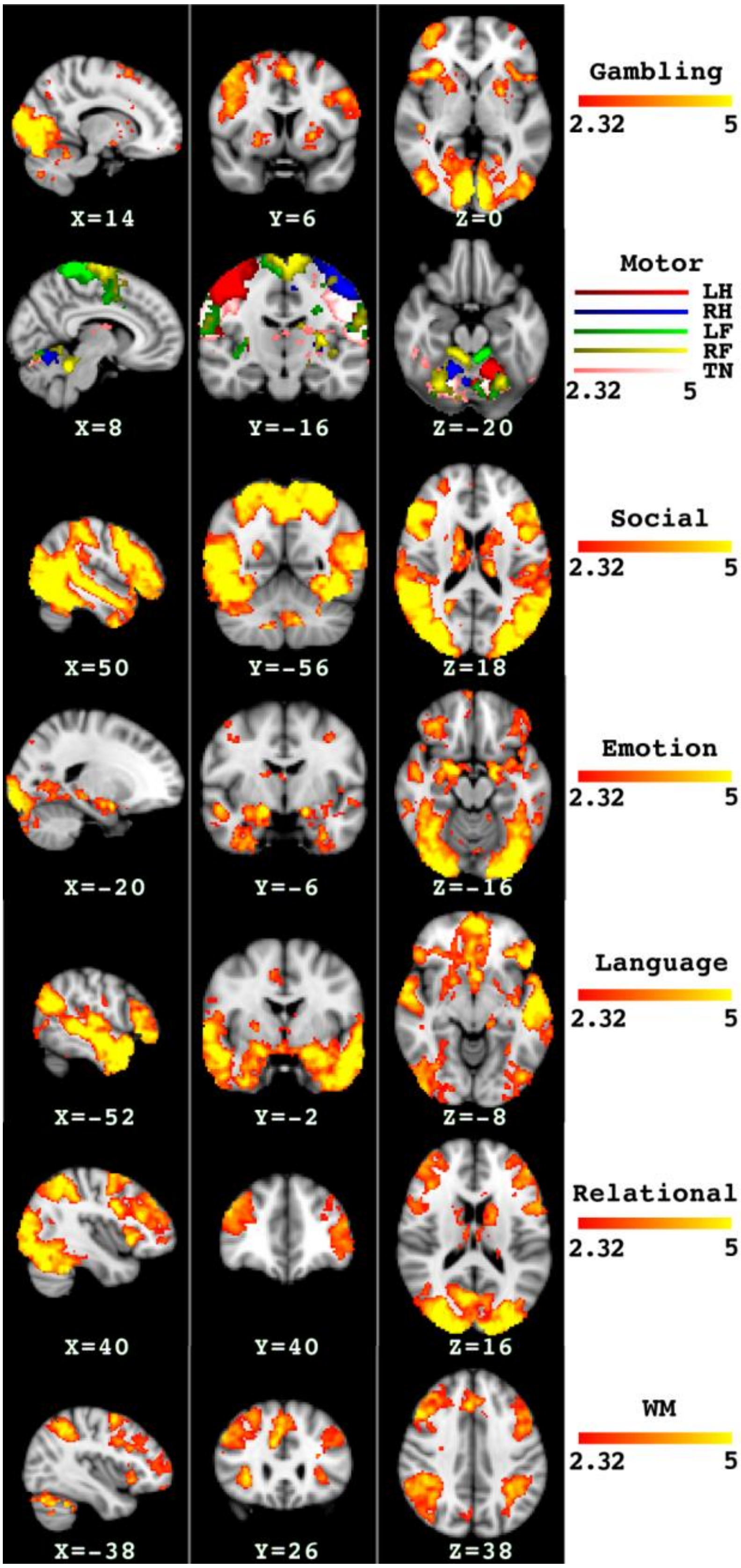

Figure S1. The activation regions for the 7 task states based on the LR dataset: (a) Emotion processing; (b) Gambling; (c) Language; (d) Motor; (e) Relational processing; (f) Social cognition; (g) Working Memory (WM). To allow for ready comparisons with the results in Barch's paper

(2013), the slices were chosen so that they would be in similar positions, and the color window is also [2.32 5]. (Throughout the manuscript, the results are presented in the same sequence in the related figures.)

In Fig. S2, we show the activation map of those contrasts not included in the manuscript (Table S2). Only 4 tasks are listed here because for the other 3 tasks either it has a single 'cope', or the combination of all "copes" has been used. For the purpose of a detailed comparison, most coordinates of the slices shown are in line with those in Barch *et al.*'s paper *(2013)*.

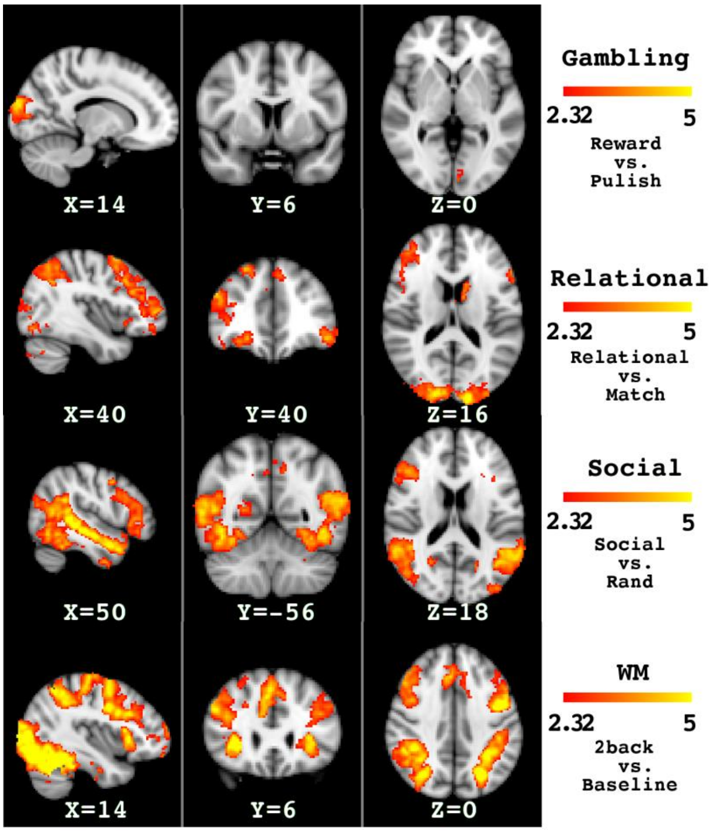

Figure S2. Other task-activation contrasts of the design matrices except for the ones used in the manuscript.

Section S3: The validation results for different thresholds and different datasets

We have 463 subjects (194 male, age 29.1 ± 3.5 years) and 465 subjects (191 male, age 29.1 ± 3.5 years) in the LR and RL dataset, respectively. For simplification, in this section, A, B and C separately indicate the thresholds of 5%, 10% and 15%. So, the label 'LR-A' means the result is based on the dataset LR with the threshold of 5%, and so forth. The results 'AVG-C' is presented in the manuscript as the our main findings.

The global efficiency comparisons between different mental states (resting and 7 tasks) (ref. Fig. 2 in the manuscript)

The efficiency indices of the whole brain in different mental states, including the resting state and the 7 tasks (gambling, motor, social cognition, emotion processing, language, and relational processing) are shown in Fig. S3. The results from the three datasets and three thresholds generated very similar trend that the global efficiency in the task states are significantly higher than the one in the resting state.

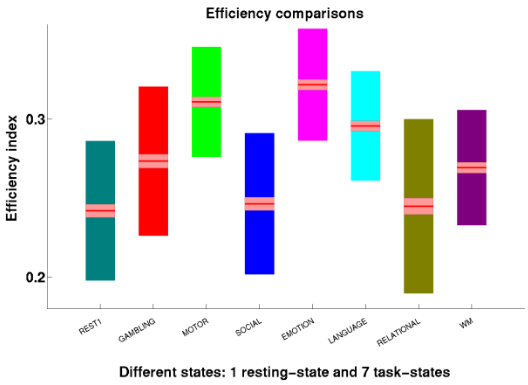

Fig. S3-AVG-A

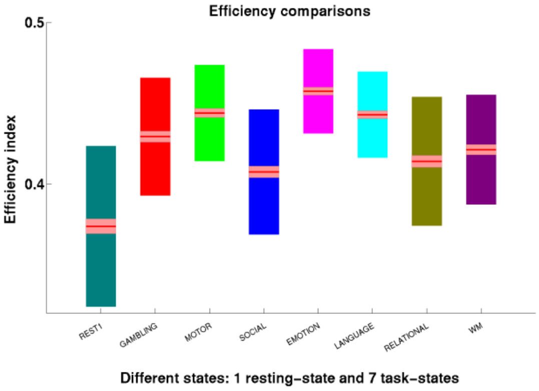

Fig. S3-AVG-B

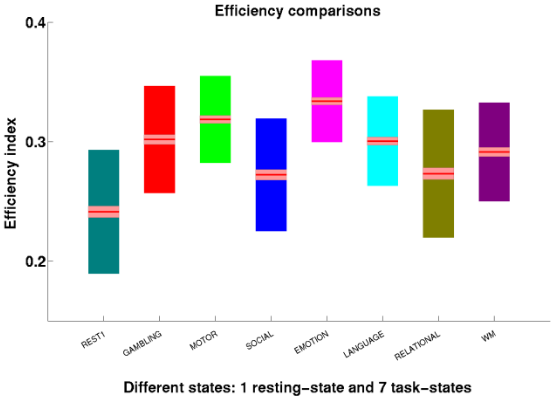

Fig. S3-LR-A

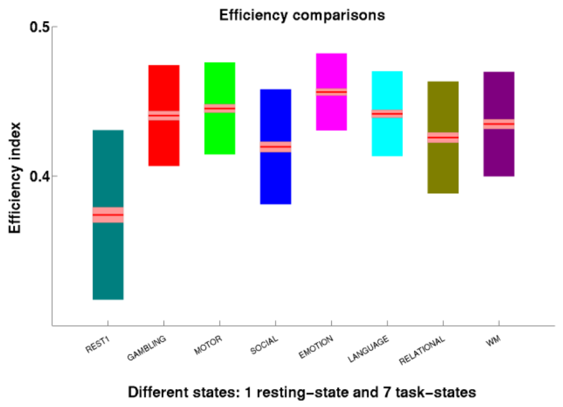

Fig. S3-LR-B

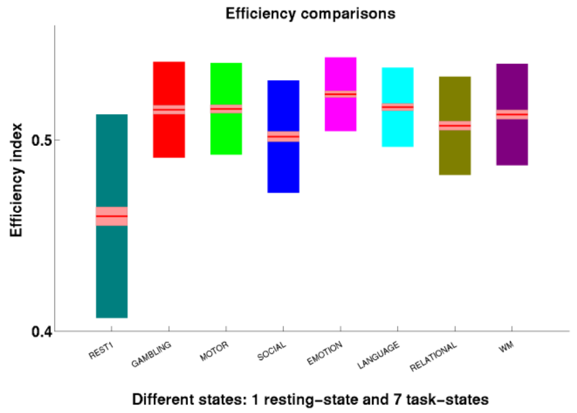

Fig. S3-LR-C

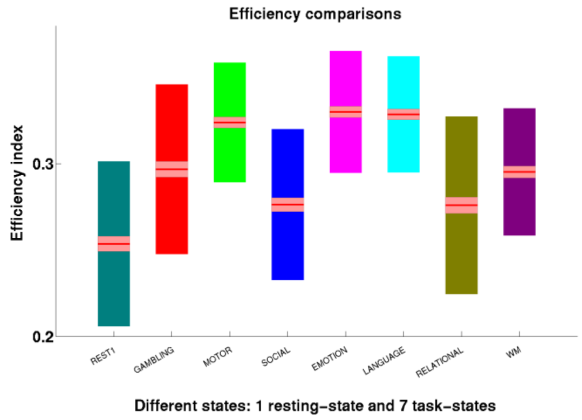

Fig. S3-RL-A

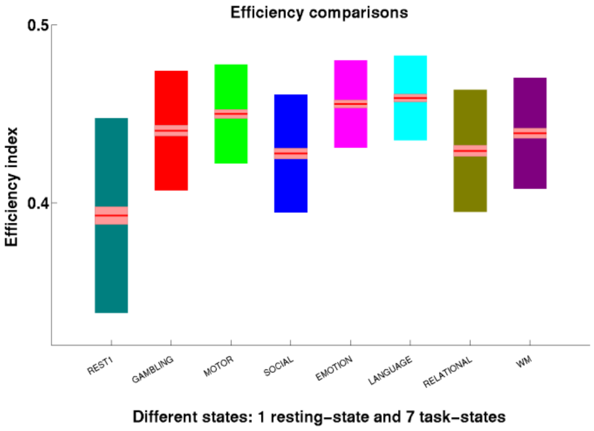

Fig. S3-RL-B

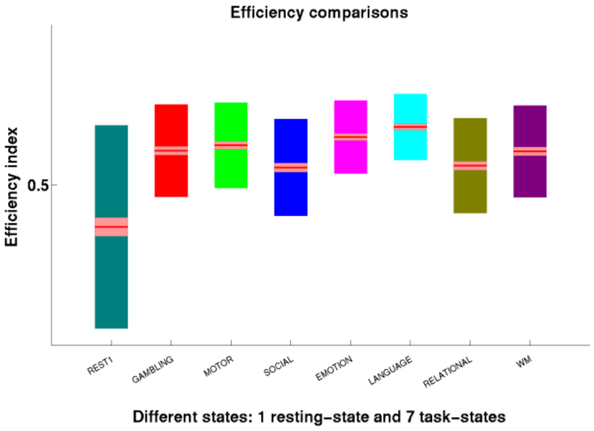

Fig. S3-RL-C

The interactions between Act/nonAct regions for the 7 resting-task pairs (ref. Fig. 4)

Comparisons of the strength of the interaction between the Act and nonAct regions in the resting and task states are shown in Fig. S4. Within each column pair, the left shows the interaction between the two classes of regions in the resting state and the right shows that in the task state. The overall results are well in line with the main findings in the manuscript except that, for the social cognition network with sparse connectivity (5% and 10% at RL dataset), the interaction between Act/nonAct regions did not decrease. However, with the increasing the density, the trend also coincides well with the main findings that the Act/nonAct interactions are increasing for the first 6 tasks but the opposite trend for the WM task.

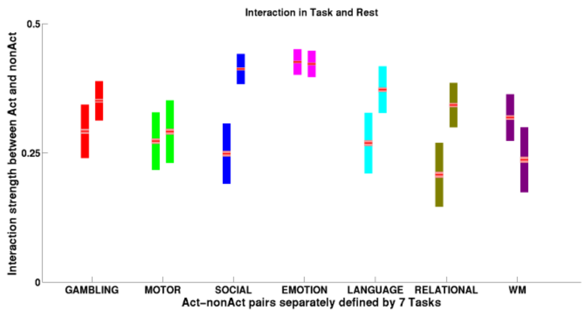

Fig. S4-AVG-A

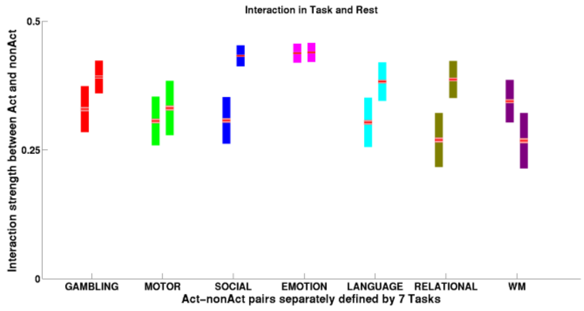

Fig. S4-AVG-B

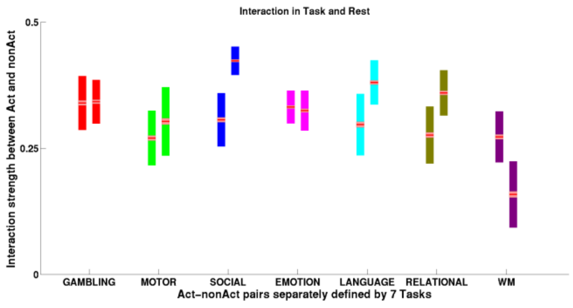

Fig. S4-LR-A

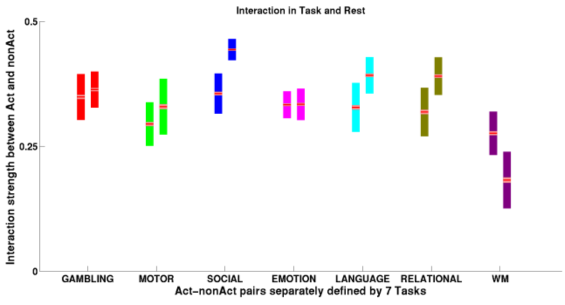

Fig. S4-LR-B

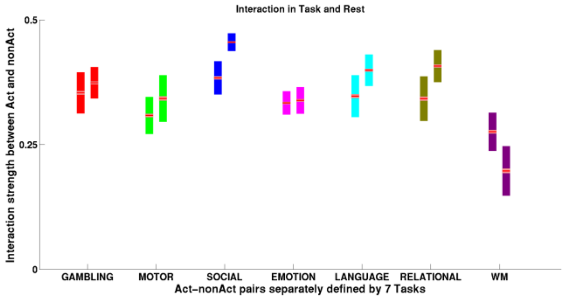

Fig. S4-LR-C

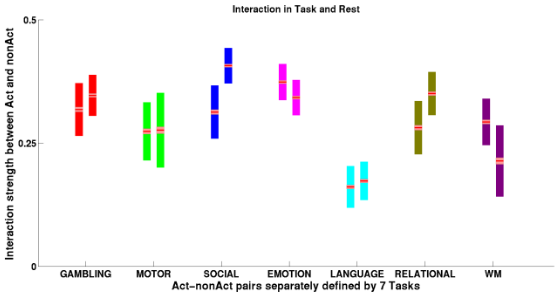

Fig. S4-RL-A

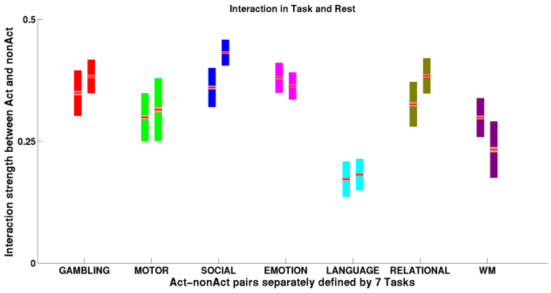

Fig. S4-RL-B

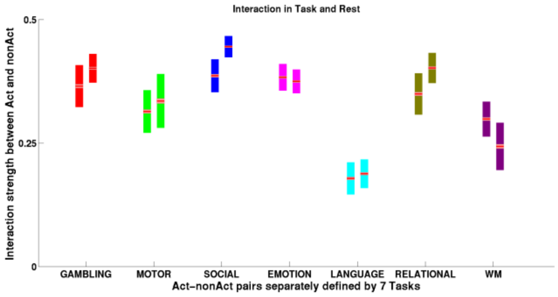

Fig. S4-RL-C

The correlations between the Act/nonAct interactions and the global efficiency (ref. Fig. 5)

Correlations between the changes in the interactions between the two regions and the changes in the global efficiency are listed in Table S3. The individual correlation strength *R* and significance level *P*-value are shown. The “thr” indicates the network density threshold.

These results collectively demonstrate that, for the first 6 resting-task comparison, the less interaction the more efficiency; and for the resting-WM comparison, the more interaction the more efficiency.

Table S3-AVG: AVG dataset

Correlations between Interaction changes and global Efficiency changes			
	thr=5 (*P*/*R*)	thr=10 (*P*/*R*)	thr=15 (*P*/*R*)
Gambling	2.28e-04/-0.17	1.36e-13/-0.34	3.48e-16/-0.37
Motor	1.08e-27/-0.48	4.31e-50/-0.62	1.08e-58/-0.66
Social	8.08e-02/0.08	5.41e-01/-0.03	1.62e-02/-0.11
Emotion	4.68e-01/-0.03	3.90e-02/-0.10	2.96e-02/-0.10
Language	6.63e-07/-0.23	1.43e-17/-0.39	1.31e-19/-0.41
Relational	2.03e-01/0.06	7.78e-09/-0.27	2.89e-16/-0.37
WM	3.33e-19/0.40	7.72e-19/0.40	1.16e-17/0.39

Table S3-LR: LR dataset

Correlations between Interaction changes and global Efficiency changes			
	thr=5 (*P*/*R*)	thr=10 (*P*/*R*)	thr=15 (*P*/*R*)
Gambling	2.50e-18/-0.39	6.57e-23/-0.44	8.70e-19/-0.40
Motor	7.03e-47/-0.60	1.74e-60/-0.67	2.08e-59/-0.66
Social	8.97e-01/-0.01	4.52e-05/-0.19	1.35e-07/-0.24
Emotion	3.62e-02/-0.10	1.35e-03/-0.15	4.27e-03/-0.13
Language	5.53e-12/-0.31	8.89e-22/-0.43	1.84e-22/-0.43
Relational	3.56e-06/-0.21	1.68e-16/-0.37	8.71e-16/-0.36
WM	2.77e-14/0.34	1.67e-18/0.39	1.08e-17/0.38

Table S3-RL: RL dataset

Correlations between Interaction changes and global Efficiency changes			
	thr=5 (*P*/*R*)	thr=10 (*P*/*R*)	thr=15 (*P*/*R*)
Gambling	3.33e-11/-0.30	1.92e-21/-0.42	2.17e-19/-0.40
Motor	4.77e-31/-0.50	1.53e-49/-0.61	7.06e-52/-0.63
Social	4.49e-02/-0.09	2.50e-10/-0.29	1.49e-12/-0.32
Emotion	4.54e-03/-0.13	3.93e-06/-0.21	8.48e-08/-0.25
Language	3.46e-07/0.23	1.88e-03/0.14	3.07e-01/0.05
Relational	3.87e-06/-0.21	1.83e-21/-0.42	2.11e-22/-0.43
WM	5.67e-25/0.45	7.16e-31/0.50	1.10e-31/0.51

The efficiency comparisons for the resting and the task (separately for the Act and nonAct) (ref. Fig. 6)

Comparisons of efficiency between the task states and resting state for Act (left panel)/nonAct (right panel) regions. The 7 column pairs in each panel are corresponding to the 7 tasks (Fig. S5). The left bar in of each column pair shows the task state and the right shows the resting state. The similar results for the Act regions do not show consistent increase trends although they have

statistically significant difference between the resting and task states.

These results collectively demonstrate that, for all the resting-task comparisons, the efficiency in Act regions significantly changes but do not show consistent trend. On the other hand, the nonAct regions shows consistently increasing efficiency.

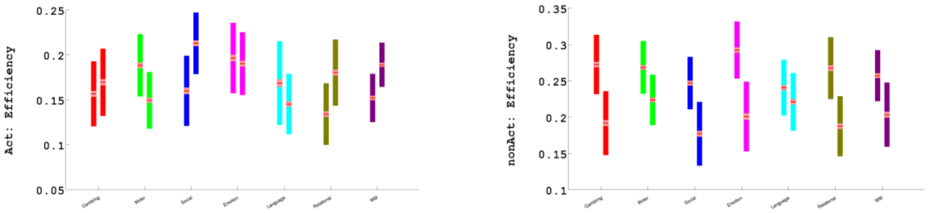

Fig. S5-AVG-A

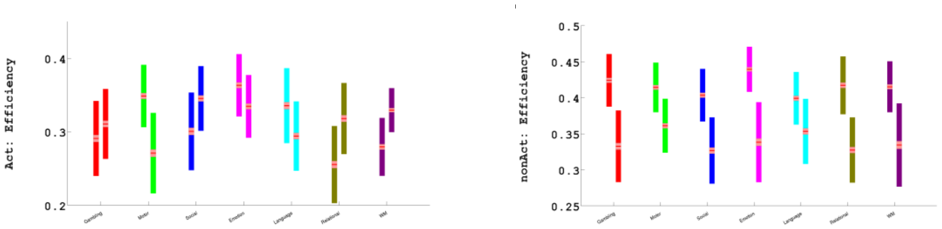

Fig. S5-AVG-B

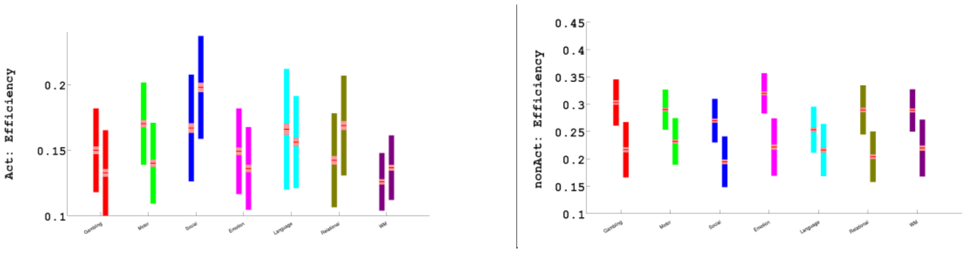

Fig. S5-LR-A

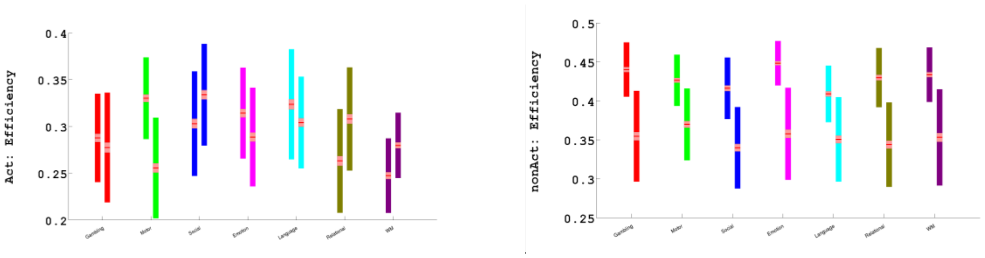

Fig. S5-LR-B

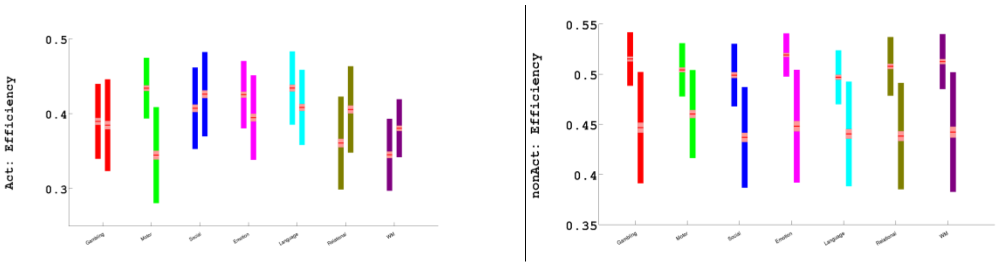

Fig. S5-LR-C

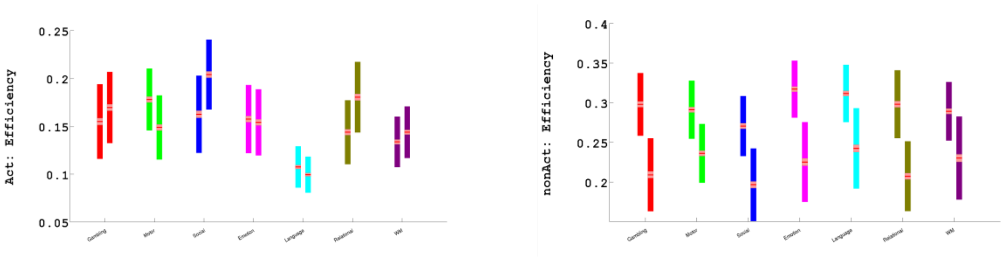

Fig. S5-RL-A

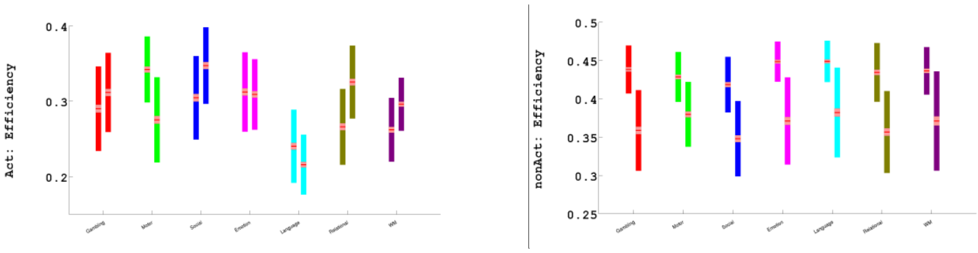

Fig. S5-RL-B

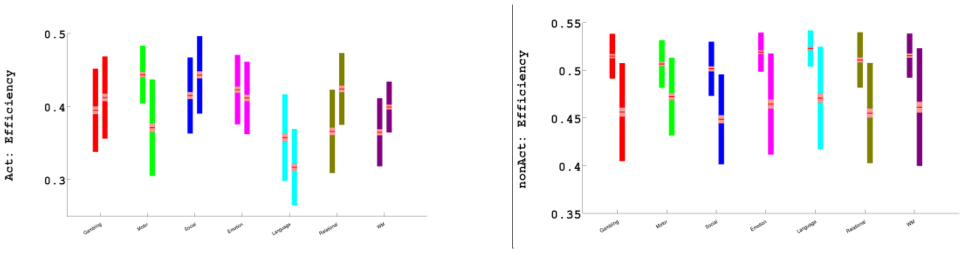

Fig. S5-RL-C

The correlations between the efficiency changes in Act/nonAct and the global efficiency (ref. Fig. 7)

Correlations between the changes in the efficiency indices between the two regions and the changes in the global efficiency are listed in Table S4. The individual correlation strength *R* and significance level *P*-value are presented. The panels A and B indicate the Act and nonAct regions, respectively.

These results consistently show that the efficiencies in Act/nonAct regions are increasing coherently with the global network efficiency.

Table S4-AVG: AVG dataset

Correlations between Efficiency changes (**Global vs. Act regions**)			
	thr=5 (P/R)	thr=10 (P/R)	thr=15 (P/R)
Gambling	1.31e-44/0.59	4.39e-59/0.66	6.81e-61/0.67

Motor	8.17e-44/0.59	7.19e-76/0.73	1.14e-98/0.79
Social	1.03e-86/0.76	1.15e-87/0.76	3.96e-92/0.78
Emotion	1.49e-44/0.59	3.89e-66/0.69	1.69e-82/0.75
Language	5.65e-13/0.33	3.52e-34/0.53	5.81e-61/0.67
Relational	4.47e-53/0.64	4.01e-65/0.69	3.62e-60/0.67
WM	1.02e-06/0.23	2.27e-09/0.28	1.07e-12/0.33

Correlations between Efficiency changes (**Global vs. nonAct regions**)			
	thr=5 (P/R)	thr=10 (P/R)	thr=15 (P/R)
Gambling	3.71e-55/0.65	3.05e-95/0.78	1.12e-136/0.86
Motor	6.36e-131/0.86	8.21e-145/0.88	7.29e-171/0.91
Social	7.83e-41/0.57	1.17e-80/0.74	5.36e-118/0.83
Emotion	1.17e-155/0.89	1.94e-225/0.95	3.28e-249/0.96
Language	3.76e-163/0.90	1.70e-173/0.91	5.89e-200/0.93
Relational	2.75e-31/0.51	4.25e-69/0.70	1.30e-111/0.82
WM	3.54e-144/0.87	8.36e-187/0.92	4.20e-213/0.94

Table S4-LR: LR dataset

Correlations between Efficiency changes (**Global vs. Act regions**)			
	thr=5 (P/R)	thr=10 (P/R)	thr=15 (P/R)
Gambling	1.50e-11/0.31	3.70e-40/0.56	1.44e-56/0.65
Motor	6.39e-25/0.45	8.34e-57/0.65	1.67e-69/0.70
Social	1.81e-68/0.70	5.60e-82/0.74	2.49e-85/0.75
Emotion	4.22e-15/0.35	8.96e-42/0.57	6.72e-63/0.68
Language	7.67e-21/0.42	1.21e-37/0.55	7.78e-60/0.66
Relational	9.45e-30/0.49	8.71e-47/0.60	1.85e-49/0.61
WM	8.96e-01/0.01	1.51e-04/0.18	2.75e-09/0.27

Correlations between Efficiency changes (**Global vs. nonAct regions**)			
	thr=5 (P/R)	thr=10 (P/R)	thr=15 (P/R)
Gambling	7.78e-136/0.86	1.86e-197/0.93	6.82e-241/0.95
Motor	3.04e-159/0.89	1.62e-208/0.93	3.54e-229/0.95
Social	7.50e-68/0.69	5.14e-117/0.83	2.89e-151/0.88
Emotion	9.65e-265/0.96	1.55e-319/0.98	0.00e+00/0.98
Language	5.05e-168/0.90	1.98e-206/0.93	1.20e-231/0.95
Relational	3.70e-54/0.64	2.41e-117/0.83	1.26e-161/0.89
WM	1.10e-238/0.95	2.17e-278/0.97	1.06e-291/0.97

Table S4-RL: RL dataset

Correlations between Efficiency changes (**Global vs. Act regions**)

	thr=5 (P/R)	thr=10 (P/R)	thr=15 (P/R)
Gambling	5.18e-30/0.49	1.17e-52/0.63	7.05e-62/0.67
Motor	1.92e-32/0.51	1.25e-59/0.66	1.19e-80/0.74
Social	5.09e-68/0.69	1.87e-80/0.74	4.89e-95/0.78
Emotion	4.54e-16/0.36	1.12e-38/0.55	8.51e-53/0.63
Language	4.34e-02/-0.09	4.80e-01/-0.03	5.72e-01/0.03
Relational	1.87e-36/0.54	2.60e-57/0.65	2.72e-56/0.65
WM	6.49e-01/0.02	5.22e-02/0.09	2.74e-03/0.14

Correlations between Efficiency changes (**Global vs. nonAct regions**)			
	thr=5 (P/R)	thr=10 (P/R)	thr=15 (P/R)
Gambling	4.01e-53/0.63	1.55e-115/0.82	1.23e-168/0.90
Motor	1.45e-136/0.86	5.23e-160/0.89	6.49e-189/0.92
Social	7.08e-58/0.65	1.60e-108/0.81	1.79e-138/0.86
Emotion	4.09e-200/0.93	1.23e-268/0.96	3.56e-293/0.97
Language	4.29e-300/0.97	0.00e+00/0.99	0.00e+00/0.98
Relational	4.39e-44/0.59	3.11e-113/0.82	4.54e-166/0.90
WM	1.96e-210/0.93	5.48e-266/0.96	1.54e-290/0.97

Section S4: The fractional modularity comparisons based on different datasets and density thresholds

When we computed the fractional modularity Q_f based on the Eq. S2 in the manuscript, the Qf was normalized by the size, namely the number of nodes for each class of regions, for a fair comparison (see Table 1). Another way for the weighting is to use all possible connections based on the nodes and this is the combination of the node, namely choosing 2 from number of nodes. After we repeated the processing in Table 1 using this weight we reproduced our findings (based on the three datasets and three thresholds, see Table S5) that from the perspective of network modularity measure, the contributions from fractional modularity of the nonAct regions is comparable to the ones from the Act regions.

Table S5-AVG: AVG dataset

**thr=15**							
Normalized by C(k,2)				Normalized by Act/nonAct size			
Both nodes (i,j) are in the examined regions		At least one node of (i,j) is in the examined regions		Both nodes (i,j) are in the examined regions		At least one node of (i,j) is in the examined regions	
reps=463	reps=7	reps=463	reps=7	reps=463	reps=7	reps=463	reps=7
F=2.24 P=0.13	F=2.24 P=0.13	F=0 P=0.93	F=0 P=0.93	F=2.52 P=0.11	F=2.51 P=0.11	F=0.95 P=0.33	F=0.95 P=0.33

thr=10

Normalized by C(k,2)				Normalized by Act/nonAct size			
Both nodes (i,j) are in the examined regions		At least one node of (i,j) is in the examined regions		Both nodes (i,j) are in the examined regions		At least one node of (i,j) is in the examined regions	
reps=463	reps=7	reps=463	reps=7	reps=463	reps=7	reps=463	reps=7
F=0.76<br>*P*=0.38	*F*=0.76<br>*P*=0.38	*F*=0.42<br>*P*=0.52	F=0.42<br>p=0.52	F=1.19<br>p=0.27	F=1.2<br>p=0.27	F=1.48<br>p=0.22	F=1.48<br>p=0.22

thr=5

Normalized by C(k,2)				Normalized by Act/nonAct size			
Both nodes (i,j) are in the examined regions		At least one node of (i,j) is in the examined regions		Both nodes (i,j) are in the examined regions		At least one node of (i,j) is in the examined regions	
reps=463	reps=7	reps=463	reps=7	reps=463	reps=7	reps=463	reps=7
F=0.7<br>P=0.4	F=0.7<br>p=0.4	F=0.13<br>p=0.72	F=0.13<br>p=0.72	F=2.02<br>p=0.16	F=2.01<br>p=0.16	F=1.54<br>p=0.21	F=1.52<br>p=0.22

Table S5-LR: LR dataset

thr=15

Normalized by C(k,2)				Normalized by Act/nonAct size			
Both nodes (i,j) are in the examined regions		At least one node of (i,j) is in the examined regions		Both nodes (i,j) are in the examined regions		At least one node of (i,j) is in the examined regions	
reps=463	reps=7	reps=463	reps=7	reps=46<br>3	reps=7	reps=46<br>3	reps=7
F=1.47<br>p=0.23	F=1.47<br>p=0.23	F=3.35<br>p=0.07	F=0.35<br>p=0.07	F=0.09<br>p=0.77	F=0.09<br>p=0.77	F=0.5<br>p=0.48	F=0.49<br>p=0.48

thr=10

Normalized by C(k,2)				Normalized by Act/nonAct size			
Both nodes (i,j) are in the examined regions		At least one node of (i,j) is in the examined regions		Both nodes (i,j) are in the examined regions		At least one node of (i,j) is in the examined regions	
reps=463	reps=7	reps=463	reps=7	reps=46<br>3	reps=7	reps=46<br>3	reps=7
F=0.49<br>p=0.48	F=0.49<br>p=0.48	F=3.15<br>p=0.08	F=3.16<br>p=0.08	F=0.49<br>p=0.49	F=0.49<br>p=0.49	F=0.02<br>p=0.9	F=0.02<br>p=0.9

thr=5

Normalized by C(k,2)	Normalized by Act/nonAct size

Both nodes (i,j) are in the examined regions		At least one node of (i,j) is in the examined regions		Both nodes (i,j) are in the examined regions		At least one node of (i,j) is in the examined regions	
reps=463	reps=7	reps=463	reps=7	reps=46 3	reps=7	reps=46 3	reps=7
F=0 p=0.99	F=0 p=0.99	F=0.26 p=0.61	F=0.27 p=0.61	F=1 p=0.32	F=1.01 p=0.32	F=0.42 p=0.51	F=0.43 p=0.51

Table S5-RL: RL dataset

thr=15

Normalized by C(k,2)				Normalized by Act/nonAct size			
Both nodes (i,j) are in the examined regions		At least one node of (i,j) is in the examined regions		Both nodes (i,j) are in the examined regions		At least one node of (i,j) is in the examined regions	
reps=463	reps=7	reps=463	reps=7	reps=463	reps=7	reps=463	reps=7
F=0.25 p=0.62	F=0.24 p=0.62	F=0.03 p=0.86	F=0.03 p=0.86	F=4.43 p=0.04	F=4.42 p=0.04	F=3.86 p=0.05	F=3.83 p=0.05

thr=10

Normalized by C(k,2)				Normalized by Act/nonAct size			
Both nodes (i,j) are in the examined regions		At least one node of (i,j) is in the examined regions		Both nodes (i,j) are in the examined regions		At least one node of (i,j) is in the examined regions	
reps=463	reps=7	reps=463	reps=7	reps=463	reps=7	reps=463	reps=7
F=0.1 p=0.75	F=0.1 p=0.75	F=0.18 p=0.67	F=0.18 p=0.67	F=0.04 p=0.85	F=0.04 p=0.85	F=0 p=0.96	F=0 p=0.96

thr=5

Normalized by C(k,2)				Normalized by Act/nonAct size			
Both nodes (i,j) are in the examined regions		At least one node of (i,j) is in the examined regions		Both nodes (i,j) are in the examined regions		At least one node of (i,j) is in the examined regions	
reps=463	reps=7	reps=463	reps=7	reps=463	reps=7	reps=463	reps=7
F=1.7 p=0.19	F=1.7 p=0.19	F=1.99 p=0.16	F=1.99 p=0.16	F=1.94 p=0.16	F=1.94 p=0.16	F=1.86 p=0.17	F=1.86 p=0.17

Section S5: The parameter settings for the generalized Louvain-based modularity computing

The Fig. 3 in the manuscript is for an overall illustration to demonstrate that the entire brain network, including both the Active and nonActive parts, reorganized the network architectures during the transition from the baseline (resting state) to each task (7 different tasks in total). In each panel in Fig. 3, it demonstrates the connectivity flow from the resting state to the task state. The modular partition is based on the generalized Louvain method *(Mucha et al., 2010)* by repetitions of 100 times to induce the random bias. The resolution parameter γ (In Eq. S2) is related to the final partition scheme through maximizing the modularity, and it has been demonstrated that for the weighted and small network the resolution influence tend to be much less significant *(Bassett et al., 2011; Good et al., 2010)*. In our study a default resolution parameter ($\gamma = 1$) was used in the Mucha's implementation *(Mucha et al., 2010)* (http://netwiki.amath.unc.edu/GenLouvain/GenLouvain). See Fig. S6 for the detailed comparisons and the rationale of the final choice. The number of modules can be affected by the resolution parameter γ and the network density. In the revised manuscript, we use the binarized association matrix and our results demonstrated comparable numbers of modules as reported in *(Power et al., 2011; Yeo et al., 2011)*. Our current modular partition derived from the weighted association matrix usually have only 2-4 main results, which largely coincides with the existing reports in Bassett's work (where the Fig. 2 used the similar methods as ours) *(Bassett et al., 2011)* and in Vatansever's work (Fig. 3 using Infomap algorithm *(Rosvall and Bergstrom, 2008)*) *(Vatansever et al., 2015)*. And this result is also well in line with Power's report (right panel in Fig. S6 using Newman's algorithm *(Newman and Girvan, 2004)*).

We further investigated the influences of on the resolution parameter γ and the number of modular partitions on the modularity computation in our manuscript. It is well known that the modularity optimization based methods are prone to be limited by the modular resolution *(Fortunato and Barthelemy, 2007)*. In our study, to make the results comparable, a unified resolution parameter is used, $\gamma = 1$, which is also the default parameter for generalized Louvain method (http://netwiki.amath.unc.edu/GenLouvain/GenLouvain). A test was performed for the influence of γ on the resting state network for subject 100307. The left panel of Fig. S6 shows the modularity indices when γ changes from 0.2 to 2.0 by step 0.2. It shows that γ=0.8 or 1.0 could achieve the maximized modularity, providing the rationale for γ= 1.0 used in our study. The right panel of Fig. S6 shows the similarity between the partition results using different γ values. The similarity was measured by the z-score of the Rand similarity coefficient *(Traud et al., 2011)*.

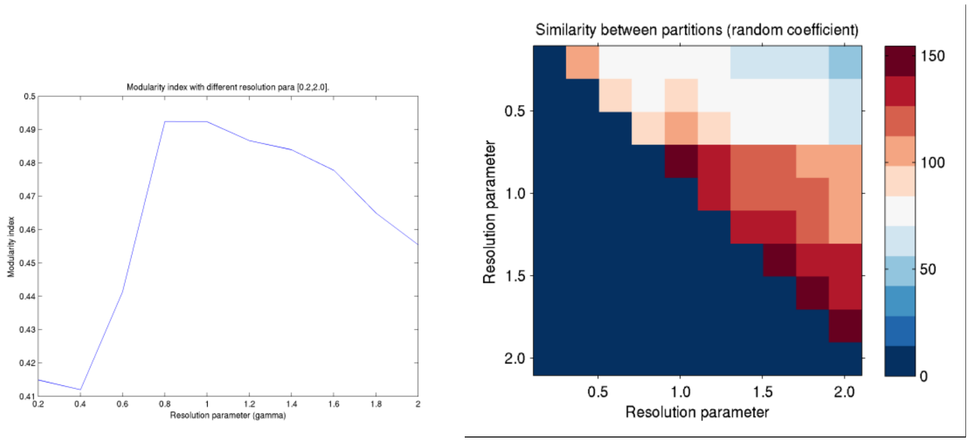

Figure S6. The influence of the different resolution parameters on the generalized Louvain modularity algorithm. The left panel shows the curve how resolution parameter influences the modularity index and the right panel shows the similarity between different partitions by different resolution parameters.

Section S6: The illustrations for the connectivity transitions between the resting state to the task states

The Fig. 3 in the manuscript showed the connectivity flow when the brain changes the state from the resting to the tasks, where the nodes were grouped as the modular assignments. Here we have generated a series of connectivity matrix maps to describe the connectivity flow between the Act and nonAct communities (see Fig. S7 below). The connectivity matrices were based on the averaged database (described above) and each matrix is the averaged connectivity for each mental state (the resting and the 7 tasks) across the 453 objects. The order for the nodes is based on the Power *et al*'s paper *(2011)*, but we grouped the nodes as Act and nonAct communities for each resting-task pair (labeled as the left vertical texts in each panel). Judging from the visual inspection, in line with the Fig. 3 in the manuscript, they consistently showed global connectivity transitions.

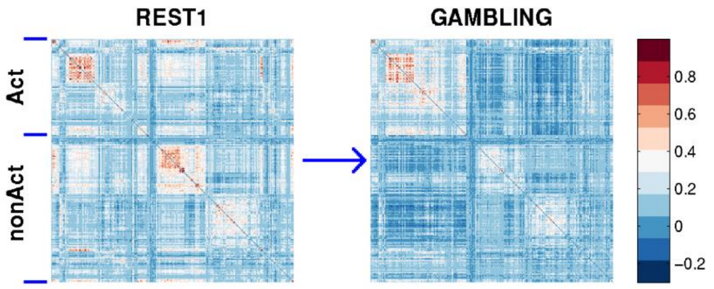

(I)

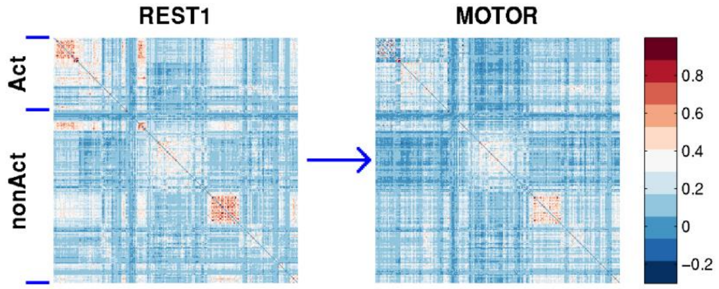
REST1
MOTOR
Act
nonAct
0.8
0.6
0.4
0.2
0
−0.2

(II)

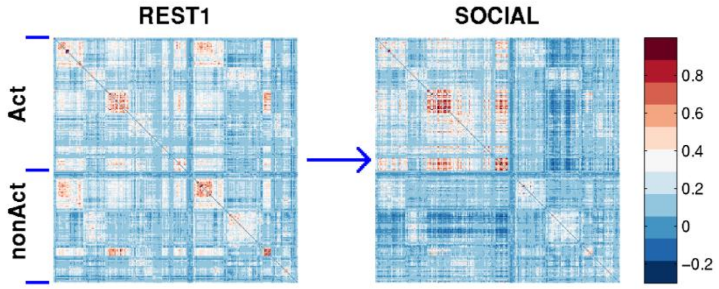
REST1
SOCIAL
Act
nonAct
0.8
0.6
0.4
0.2
0
−0.2

(III)

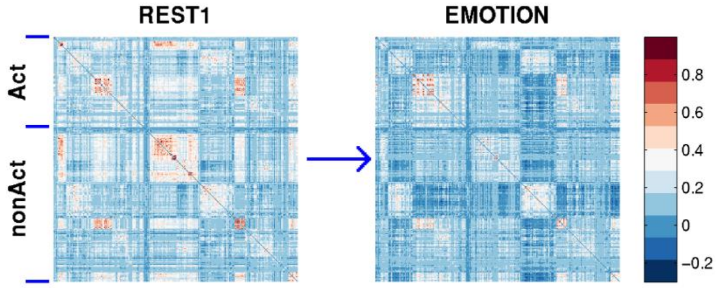
REST1
EMOTION
Act
nonAct
0.8
0.6
0.4
0.2
0
−0.2

(IV)

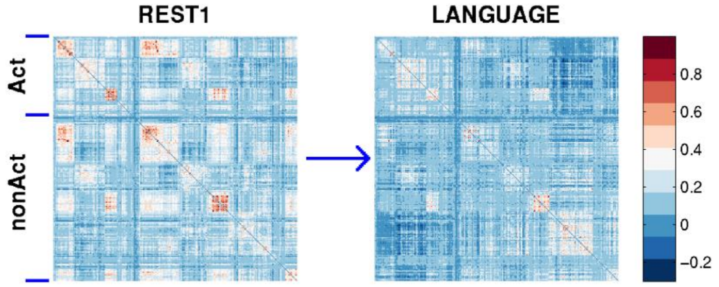
REST1
LANGUAGE
Act
nonAct
0.8
0.6
0.4
0.2
0
−0.2

(V)

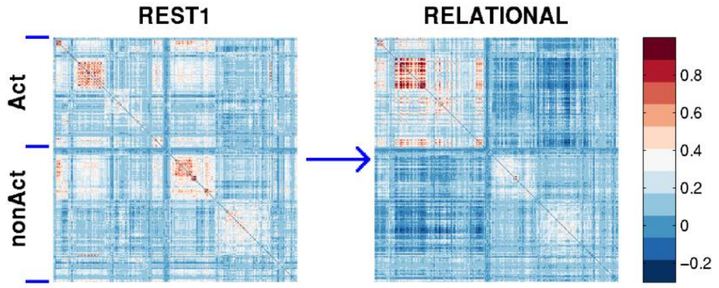
REST1
RELATIONAL
Act
nonAct
0.8
0.6
0.4
0.2
0
−0.2

(VI)

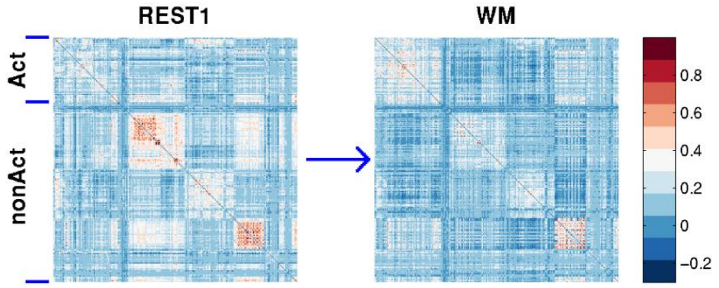

(VII)

Figure S7. The illustrations for the functional connectivity transitions from the baseline (resting state) to the 7 tasks, where panel I-VII separately indicate the 7 resting-task pairs.

Section S7: Methodological considerations

Definitions of the Act and nonAct regions. In this study, the investigation into the interactions between the Act and nonAct regions was based on an activation detection strategy, which depends on several factors, including the regression methods, contrast design, and the threshold at the participant and group levels. We utilized a traditional GLM model implemented in FSL *(Smith et al., 2004)*, which can be used to generate a generally accepted activation map for the 7 task states *(Barch et al., 2013)*. Two additional factors, setting an appropriate task contrast (see Table 1) and reserving the regions with a considerable size to avoid too few ROIs remaining in the activation regions, were utilized to seek a balance between sensitivity and specification. To this end, the task contrasts were set as in Table 1 with Z = 1.96 and P = 0.05 for the individual participants and Z = 2.32 and P = 0.05 at the group level in the FSL/FEAT pipeline. In Section 1 of the Supplementary Material, for a better comparison, we listed the activation detection results from different task-baseline contrasts. It should be noted that both different task contrasts and different parameter settings in activation detection could result in different activation maps and different patterns of functional connectivity for the Active and nonAct regions. The activation detection strategy in our study is one of the mostly used ones, and the activation regions are well accepted in the literature for the specific tasks *(Barch et al., 2013)*. Therefore, it is unnecessary, if not impossible, to enumerate all the possible combinations of contrasts and parameter settings for robust determination of the Act and nonAct regions. From this view, our study could shed light on the collaborations between the Active and nonAct regions. The final activation masks adopted in this work are also available for download.

Construction of functional brain networks. Since different strategies to measure the functional connectivity and then construct a network exist, in this study, for a complete validation of our findings, three connectivity density thresholds, 5%, 10% and 15%, have been considered *(Bullmore and Sporns, 2009; Rubinov and Sporns, 2010; van Wijk et al., 2010)* to construct the functional network separately in three datasets, LR phase encoding direction, RL phase encoding direction and the averaged connectivity after the Fisher z-transform. Moreover, before calculating the functional connectivity for the task fMRI time series, the mean task activity has been regressed out and the residuals were used in constructing networks *(Cole et al., 2013)*.

Modularity and efficiency computing. Modularity and efficiency were the two main measures in this study that were used to characterize the global and local (within one class of regions) network evolvement. An extended optimization-based Louvain method was used in this study (*Mucha et al., 2010*). In addition, a fractional modularity measure was adopted to compare the contributions from the Act and the nonAct regions to the global changes in the modularity separately. The modularity index focuses on the functional segregation property of a network, and the efficiency index was used to characterize the information communicating efficiency of two regions (*Latora and Marchiori, 2003*; *Rubinov and Sporns, 2010*). Therefore, the regions assignment and the regions between the Act and nonAct regions of the network have been considerably characterized in the current study.

Appendix S1: The 30 subjects for generating Act and nonAct regions masks

The unrelated subjects were chosen by several criteria: 1. They do not have twins or shared parents in the dataset; 2. Aged between 20-30. The 30-subjects list used in this study is as follows.

101107
102008
105216
113215
118730
118932
122620
123420
130013
135225
144832
147030
148840
150726
151223
153833
157437
162228
167743
172534
168139
173435
173940
178849

180129
187143
204016
208226
210011
214423

Appendix S2: The masks for the Act regions (hence the rest of the brain is the nonAct regions)

The activation masks (Act regions) for 7 tasks used in this study are available in the following link, which are generated by the FSL/FEAT.
https://github.com/nmzuo/Act-L-Act-network .

References

[1]. Barch D.M., Burgess G.C., Harms M.P., Petersen S.E., Schlaggar B.L., Corbetta M., et al., Function in the human connectome: task-fMRI and individual differences in behavior. *Neuroimage*, 2013. 80, 169-189.

[2]. Bassett D.S., Wymbs N.F., Porter M.A., Mucha P.J., Carlson J.M., Grafton S.T., Dynamic reconfiguration of human brain networks during learning. *Proc Natl Acad Sci U S A*, 2011. 108, 7641-7646.

[3]. Bassett D.S., Yang M., Wymbs N.F., Grafton S.T., Learning-induced autonomy of sensorimotor systems. *Nat Neurosci*, 2015. 18, 744-751.

[4]. Beckmann C.F., Jenkinson M., Smith S.M., General multilevel linear modeling for group analysis in FMRI. *Neuroimage*, 2003. 20, 1052-1063.

[5]. Bianciardi M., Fukunaga M., van Gelderen P., de Zwart J.A., Duyn J.H., Negative BOLD-fMRI signals in large cerebral veins. *J Cereb Blood Flow Metab*, 2011. 31, 401-412.

[6]. Bullmore E., Sporns O., Complex brain networks: graph theoretical analysis of structural and functional systems. *Nat Rev Neurosci*, 2009. 10, 186-198.

[7]. Cohen J.D., Perlstein W.M., Braver T.S., Nystrom L.E., Noll D.C., Jonides J., et al., Temporal dynamics of brain activation during a working memory task. *Nature*, 1997. 386, 604-608.

[8]. Cole M.W., Reynolds J.R., Power J.D., Repovs G., Anticevic A., Braver T.S., Multi-task connectivity reveals flexible hubs for adaptive task control. *Nat Neurosci*, 2013. 16, 1348-1355.

[9]. Fortunato S., Barthelemy M., Resolution limit in community detection. *Proc Natl Acad Sci U S A*, 2007. 104, 36-41.

[10]. Glasser M.F., Sotiropoulos S.N., Wilson J.A., Coalson T.S., Fischl B., Andersson J.L., et al., The minimal preprocessing pipelines for the Human Connectome Project. *Neuroimage*, 2013. 80, 105-124.

[11]. Good B.H., de Montjoye Y.A., Clauset A., Performance of modularity maximization in practical contexts. *Phys Rev E Stat Nonlin Soft Matter Phys*, 2010. 81, 046106.

[12]. Hu D., Huang L., Negative hemodynamic response in the cortex: evidence opposing neuronal deactivation revealed via optical imaging and electrophysiological recording. *J Neurophysiol*, 2015. 114, 2152-2161.

[13]. Jenkinson M., Beckmann C.F., Behrens T.E., Woolrich M.W., Smith S.M., Fsl. *Neuroimage*, 2012. 62, 782-790.

[14]. Latora V., Marchiori M., Economic small-world behavior in weighted networks. *Eur Phys J B*, 2003. 32, 249-263.

[15]. Mayhew S.D., Mullinger K.J., Ostwald D., Porcaro C., Bowtell R., Bagshaw A.P., et al., Global signal modulation of single-trial fMRI response variability: Effect on positive vs negative BOLD response relationship. *Neuroimage*, 2016. 133, 62-74.

[16]. Mucha P.J., Richardson T., Macon K., Porter M.A., Onnela J.P., Community structure in time-dependent, multiscale, and multiplex networks. *Science*, 2010. 328, 876-878.

[17]. Newman M.E., Girvan M., Finding and evaluating community structure in networks. *Phys Rev E Stat Nonlin Soft Matter Phys*, 2004. 69, 026113.

[18]. Power J.D., Cohen A.L., Nelson S.M., Wig G.S., Barnes K.A., Church J.A., et al., Functional network organization of the human brain. *Neuron*, 2011. 72, 665-678.

[19]. Rosvall M., Bergstrom C.T., Maps of random walks on complex networks reveal community structure. *Proc Natl Acad Sci U S A*, 2008. 105, 1118-1123.

[20]. Rubinov M., Sporns O., Complex network measures of brain connectivity: uses and interpretations. *Neuroimage*, 2010. 52, 1059-1069.

[21]. Shih Y.Y., Chen C.C., Shyu B.C., Lin Z.J., Chiang Y.C., Jaw F.S., et al., A new scenario for negative functional magnetic resonance imaging signals: endogenous neurotransmission. *J Neurosci*, 2009. 29, 3036-3044.

[22]. Smith S.M., Jenkinson M., Woolrich M.W., Beckmann C.F., Behrens T.E., Johansen-Berg H., et al., Advances in functional and structural MR image analysis and implementation as FSL. *Neuroimage*, 2004. 23 Suppl 1, S208-219.

[23]. Traud A.L., Kelsic E.D., Mucha P.J., Porter M.A., Comparing Community Structure to Characteristics in Online Collegiate Social Networks. *Siam Review*, 2011. 53, 526-543.

[24]. Van Essen D.C., Smith S.M., Barch D.M., Behrens T.E., Yacoub E., Ugurbil K., et al., The WU-Minn Human Connectome Project: an overview. *Neuroimage*, 2013. 80, 62-79.

[25]. van Wijk B.C., Stam C.J., Daffertshofer A., Comparing brain networks of different size and connectivity density using graph theory. *PLoS One*, 2010. 5, e13701.

[26]. Vatansever D., Menon D.K., Manktelow A.E., Sahakian B.J., Stamatakis E.A., Default Mode Dynamics for Global Functional Integration. *J Neurosci*, 2015. 35, 15254-15262.

[27]. Woolrich M.W., Ripley B.D., Brady M., Smith S.M., Temporal autocorrelation in univariate linear modeling of FMRI data. *Neuroimage*, 2001. 14, 1370-1386.

[28]. Yeo B.T., Krienen F.M., Sepulcre J., Sabuncu M.R., Lashkari D., Hollinshead M., et al., The organization of the human cerebral cortex estimated by intrinsic functional connectivity. *J Neurophysiol*, 2011. 106, 1125-1165.

[29]. Zuo N., Song M., Fan L., Eickhoff S.B., Jiang T., Different interaction modes for the default mode network revealed by resting state functional magnetic resonance imaging. *Eur J Neurosci*, 2016. 43, 78-88.
